# Age differences in psychological comorbidity in atopic dermatitis: a systematic review and meta-analysis

**DOI:** 10.3389/fpubh.2026.1818252

**Published:** 2026-07-07

**Authors:** Hongli Wang, Huike Ma, Dairong Wei, Yirong Xi, Jingxia Zhao

**Affiliations:** Beijing Hospital of Traditional Chinese Medicine, Capital Medical University, Beijing Institute of Chinese Medicine, Beijing, China

**Keywords:** adult, anxiety, atopic dermatitis, child, depression, mental disorders

## Abstract

**Background:**

While the association between atopic dermatitis (AD) and psychological comorbidities is established, whether this risk differs across developmental stages remains unclear. This study aimed to quantify and compare this risk and examine key methodological sources of heterogeneity.

**Methods:**

We conducted a systematic search of PubMed, Embase, and Cochrane Library for studies published up to October 2025. Pooled effect estimates [hazard ratios (HRs) from cohort/case-control studies and odds ratios (ORs) from cross-sectional studies] were calculated using random-effects models. Ninety-five percent prediction intervals were calculated for all meta-analyses. The study protocol was registered with PROSPERO (CRD420251244849).

**Results:**

We included 28 studies of various observational designs (cohort, cross-sectional, and case-control) in the meta-analysis. The analysis revealed numerically higher risk estimates for depression, anxiety, and suicidality among pediatric and adolescent AD patients compared with adults. Specifically, for depression, the pooled HR from cohort studies in youth was 2.16 (95% CI: 1.56–2.98; *I*^2^=99.1%, *P* < 0.001) versus 1.32 (95% CI: 1.23–1.41; *I*^2^ = 94.9%, *P* < 0.001) in adults; similarly, the pooled OR from cross-sectional studies in youth was 2.79 (95% CI: 1.52–5.12; *I*^2^ = 99.4%, *P* < 0.001) versus 1.61 (95% CI: 1.22–2.14; *I*^2^ = 92.4%, *P* < 0.001) in adults. A similar pattern was observed for anxiety. Additionally, younger patients exhibited an elevated risk of suicide relative to adults.

**Conclusion:**

The psychological burden of AD appears most pronounced in children and adolescents, but the very high heterogeneity across studies limits the certainty of this finding. Therefore, the results should be interpreted cautiously.

**Systematic review registration:**

https://www.crd.york.ac.uk/prospero/display_record.php?RecordID=1244849, identifier: CRD420251244849.

## 1 Introduction

Atopic dermatitis (AD) is a chronic, non-contagious inflammatory skin disease characterized by persistent pruritus (1). The chronic relapsing course of the disease, its economic burden, and the involvement of the whole family in the management process significantly impair the quality of life of patients and their families, affecting a large number of pediatric and adult patients worldwide and causing severe, multidimensional damage to patient wellbeing (2). Beyond the prominent physical symptoms, the close association between AD and a range of psychological comorbidities has attracted increasing attention. Studies consistently indicate that AD patients have a significantly higher risk of developing psychological disorders such as depression and anxiety compared to the general population (3). Potential mechanisms operate across biopsychosocial domains, ranging from the chronic itch-scratch cycle and sleep disruption to social stigma and dysregulation of shared neuro-immune pathways (4, 5).

Although existing research has confirmed the association between AD and psychological disorders, the considerable heterogeneity in the strength of this association poses a significant challenge for informing public health practice (6). This inconsistency, particularly stemming from differences across age groups, makes it difficult to accurately estimate the true burden of psychological disorders in the AD population. Consequently, it hinders the development of age-specific, targeted screening strategies and the effective allocation of intervention resources. To address this critical evidence gap, we conducted an age-stratified systematic review and meta-analysis. The primary objective was to quantify and compare the pooled risk of psychological disorders (such as depression and anxiety) between pediatric/adolescent and adult AD patients, thereby delineating an age-specific risk profile. The findings are expected to provide direct evidence for establishing development-stage-appropriate, evidence-based guidelines for managing psychological comorbidities in AD, promoting a shift in public health practice from a “one-size-fits-all” to a “stratified-precision” paradigm.

## 2 Methods

### 2.1 Search strategy

This meta-analysis was conducted in accordance with the Preferred Reporting Items for Systematic Reviews and Meta-Analyses (PRISMA) 2020 guidelines (7). The study protocol was registered with CRD420251244849 and is accessible at: https://www.crd.york.ac.uk/prospero/display_record.php?RecordID=1244849.

### 2.2 Data sources

A comprehensive literature search was conducted in PubMed, Cochrane Library, and Embase databases from their inception until October 18, 2025. The search strategy combined both controlled vocabulary and free-text keywords to identify relevant studies. Key search terms encompassed concepts related to atopic dermatitis (e.g., atopic dermatitis, eczema, chronic atopy) and psychological/neuropsychiatric comorbidities (e.g., neuropsychiatric disorder, mental disease, autism, attention deficit hyperactivity disorder, cognitive impairment). The detailed search strategies for each database are provided in Supplementary Tables 1–3.

### 2.3 Eligibility criteria

The studies included in this analysis fulfilled the following eligibility criteria: (1) Population: The study population comprised patients diagnosed with AD according to established diagnostic criteria or physician diagnosis, with explicit stratification or reporting of outcomes separately for children/adolescents (typically < 18 years) and adults (≥18 years). (2) Exposure: Diagnosis of AD. (3) Comparator: Healthy controls, general population without AD, or within-study comparisons across different age groups in AD patients. (4) Outcomes: Prevalence, incidence, hazard ratios (HR), odds Ratio (OR) or risk estimates of psychological comorbidities (e.g., depression, anxiety). (5) Study Design: Eligible studies include cohort studies, cross-sectional studies. (6) Exclusion Criteria: Reviews, meta-analyses, Mendelian randomization studies, duplicate publications, studies with incomplete outcome data or irrelevant outcomes, and non-English studies without available translation.

### 2.4 Study selection

Two reviewers (Huike Ma and Jingxia Zhao) independently screened studies based on the predefined eligibility criteria. After removing duplicates, they reviewed titles and abstracts to exclude clearly irrelevant records. The remaining potentially eligible articles underwent full-text assessment. Disagreements at any stage were resolved through discussion, and unresolved cases were adjudicated by a senior investigator (Hongli Wang) to reach a final decision.

### 2.5 Data extraction

Four researchers (Hongli Wang, Huike Ma, Dairong Wei, and Yirong Xi) collaboratively developed a standardized data extraction form in Microsoft Excel. The extracted information included the first author, year of publication, country of origin, participant age, and diagnostic criteria for AD, among others. All data were extracted independently by two reviewers, cross-verified, and any discrepancies were resolved through group discussion to reach consensus.

### 2.6 Quality assessment and risk of bias

We assessed methodological quality using two versions of the Newcastle-Ottawa Scale (NOS): the standard NOS for cohort and case-control studies (8), and the NOS-xs (a modified version for cross-sectional studies adapted from Carra MC et al. (9)). The NOS-xs employs a nine-star scoring system and evaluates six items across three domains: sample selection, exposure/outcome assessment, and confounding factors. Regardless of study design, studies were classified as low (0–3 stars), moderate (4–6 stars), or high quality (7–9 stars). Two reviewers independently assessed each study, and any disagreements were resolved by consensus.

### 2.7 Statistical analysis

We extracted adjusted HRs from cohort/case-control studies and ORs from cross-sectional studies. No direct pooling of HRs and ORs was performed; separate meta-analyses were conducted for each effect measure type, and no effect measure conversions were applied. Between-study heterogeneity was quantified using *I*^2^, and a random-effects model was used when *I*^2^ > 50%. Given the high heterogeneity (*I*^2^ > 90% for most outcomes), we calculated 95% prediction intervals (PIs) for any meta-analysis with at least three studies (k ≥ 3) (8, 9). Leave-one-out sensitivity analysis was performed. Publication bias was not formally tested because for most analyses *k* < 10, where funnel plot asymmetry tests have low power and can be misleading. Additionally, high heterogeneity itself may induce asymmetry unrelated to publication bias. All analyses were conducted in Stata 17.0.

## 3 Results

### 3.1 Literature search

**Figure 1:**
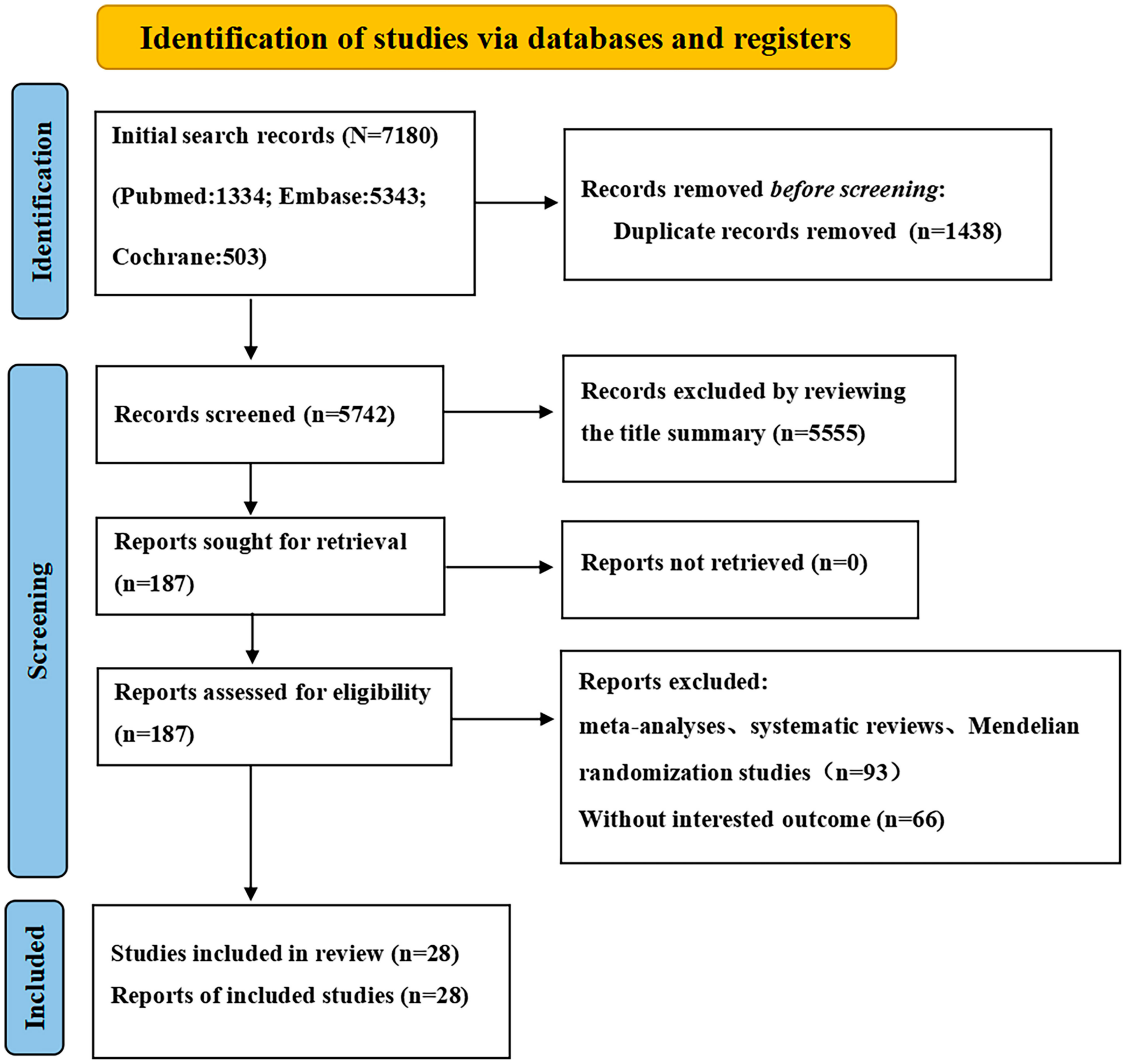
Studies screening process. PRISMA flow diagram of the screening and selection process according to PRISMA 2020 guidelines.

A systematic search conducted up to October 18, 2025, initially yielded 7,180 records. After removing duplicates (*n* = 1,438), 5,742 records remained. Based on title and abstract screening, 5,555 articles were excluded as irrelevant. The remaining 187 studies underwent full-text review, from which 28 studies met the eligibility criteria (cohort, cross-sectional, and case-control designs). All included studies reported associations between AD and psychological comorbidities. The detailed study selection process is illustrated in Figure 1.

### 3.2 Study characteristics

This meta-analysis ultimately included 28 studies published in the past decade, comprising 10 cohort studies, 3 case-control studies, and 15 cross-sectional studies. Among them, 10 studies focused on adult populations (10–19), 16 studies targeted children/adolescents (20–35), and 2 studies covered both age groups (36, 37). These studies were conducted across multiple countries and regions, including the United States (15 studies), the United Kingdom (2 studies), China (2 studies), South Korea (3 studies), Germany (2 studies), as well as Denmark, Switzerland, Spain, and the Netherlands (1 study each). Detailed characteristics of the included studies are presented in Table 1.

**Table 1:** Characteristics of studies on psychological comorbidity and atopic dermatitis.

Reference	Country	AD patients, *n*	Non–AD, *n*	Study design	Outcome	Age, years
Singhal et al. (19)	UK	267,788	Not specified	Cohort study	Suicide attempts	Adults (≥18)
Kim et al. (17)	Korea	434	23,008	Cross–sectional study	Depression	Adults (≥19)
Yu et al. (10)	US	109	3,616	Cross–sectional study	Depression; Sleep impairment; Suicide attempts; Eating disorder	Adults (≥18)
Cheng et al. (37)	China	8,208	8,208	Cohort study	Anxiety; Depression	Both (32.6 ± 16.1)
Lee et al. (35)	China	18,473	18,473	Case–control study	ADHD; Autism	Children (< 18)
Strom et al. (36)	US	33,865	316,456	Cross–sectional study	ADHD	Both (2+)
Lee and Shin (32)	Korea	3,091	46,914	Cross–sectional study	Suicide attempts; Depression	Children (12–17)
McKenzie and Silverberg (31)	US	1,603	11, 658	Cross–sectional study	Autism; Depression; Anxiety; Stress	Children (< 17)
Silverberg et al. (15)	US	602	1, 471	Cross–sectional study	Depression; Anxiety	Adults (≥18)
Theodosiou et al. (16)	Switzerland	602	1,471	Cross–sectional study	Anxiety; Depression	Adults (≥18)
Cheng and Silverberg (14)	US	4,175	30,138	Cross–sectional study	Sleep impairment; Anxiety; Depression	Adults (≥18)
Schonmann et al. (12)	US	436,918	2,341,285,705	Cohort study	Depression; Nervousness	Adults (≥18)
Teichgräber et al. (34)	Germany	526,808	2,569,030	Cohort study	Anxiety; Depression	Children (5–17)
Kyung et al. (28)	Korea	7,061	7,061	Case–control study	Depression	Children (13–18)
Gilaberte et al. (29)	Spain	15,536	46,740	Cross–sectional study	Depression; Suicide attempts; Stress	Children (12–18)
Wan et al. (30)	US	33,591	182,700	Cross–sectional study	ADHD	Children (< 18)
Jackson–Cowan et al. (24)	US	6,807,687	50,919,169	Cross–sectional study	ADHD	Children (< 18)
Cheng et al. (23)	US	13,398	96,084	Cross–sectional study	ADHD	Children (5–17)
Fishbein et al. (20)	US	22,716,193	815,052,954	Cross–sectional study	Anxiety; Depression; Nervousness	Children (5–17)
Kern et al. (25)	US	180	1,545	Cross–sectional study	Sleep impairment; Anxiety; Depression; Fatigue	Children (5–15)
Manjunath and Silverberg (26)	UK	4,456	6,725	Cohort study	Depression	Children (5–15)
Vittrup et al. (27)	Denmark	1,693	8,484	Cohort study	Sleep impairment; ADHD; Anxiety; Depression	Children (10–18)
Fan et al. (22)	US	14,283	142,830	Cohort study	ADHD; Anxiety; Depression; Suicide attempts	Children (< 18)
Wan et al. (21)	US	11,752	47,008	Case–control study	ADHD	Adults (≥18)
Devjani et al. (13)	US	625,083	2,678,888	Cohort study	ADHD; Anxiety; Depression; Suicide; attempts; Schizophrenia; Bipolar disorder	Adults (≥18)
Zhang et al. (18)	Netherlands	69	403	Cross–sectional study	Anxiety; Panic	Adults (20–39)
Wan et al. (21)	US	5,196	51,174	Cohort study	Anxiety; Depression; ADHD; Obsessive–compulsive disorder; Panic; Eating disorder	Children (< 18)
Mann et al. (33)	Germany	409,431	1,809,029	Cohort study	ADHD; Anxiety; Depression; Autism	Children (5–17)

### 3.3 Quality assessment

In this study, the Newcastle-Ottawa Scale (NOS) was used to assess the methodological quality of 13 cohort/case-control studies, while the NOS-xs (a modified version of the NOS adapted for cross-sectional studies) was used to assess 15 cross-sectional studies. The quality classification criteria were uniformly applied as follows: 0–3 stars indicated low quality, 4–6 stars indicated moderate quality, and 7–9 stars indicated high quality.

Among the 13 cohort/case-control studies, total NOS scores ranged from 6 to 8 stars. Specifically, 4 studies (30.8%) received 6 stars (moderate quality), 8 studies (61.5%) received 7 stars, and 1 study (7.7%) received 8 stars (high quality). All studies scored ≥6 stars, with 30.8% classified as moderate quality and 69.2% as high quality. No study was rated as low quality.

Among the 15 cross-sectional studies, total NOS-xs scores also ranged from 6 to 8 stars. Specifically, 4 studies (26.7%) received 6 stars (moderate quality), 7 studies (46.7%) received 7 stars, and 4 studies (26.7%) received 8 stars (high quality). All cross-sectional studies scored ≥6 stars, with 26.7% classified as moderate quality and 73.3% as high quality. No study was rated as low quality.

In summary, the 28 included studies demonstrated generally high methodological quality, with all studies rated as either moderate or high quality. This supports the credibility of the existing evidence in this field. Detailed scoring results are presented in Table 2.

**Table 2:** The Newcastle–Ottawa scale.

Cohort/case–control studies				
**Reference**	**Selection**	**Comparability**	**Outcome**	**Total**
Mann (33)	^**^	^**^	^**^	6
Wan et al. (21)	^***^	^**^	^***^	8
Zhang et al. (18)	^**^	^**^	^***^	7
Fan et al. (22)	^**^	^**^	^**^	6
Manjunath and Silverberg (26)	^**^	^**^	^***^	7
Vittrup et al. (27)	^**^	^**^	^***^	7
Teichgräber et al. (34)	^**^	^**^	^**^	6
Schonmann et al. (12)	^**^	^**^	^***^	7
Cheng and Silverberg (14)	^**^	^**^	^***^	7
Kern et al. (25)	^***^	^**^	^**^	7
Lee et al. (35)	^**^	^**^	^***^	7
Cheng et al. (37)	^**^	^**^	^***^	7
Singhal et al. (19)	^**^	^**^	^**^	6
**Cross–sectional studies**				
**Reference**	**Study sample selection**	**Assessment of exposure (s) and outcome (s)**	**Confounding factors**	**Total**
Devjani et al. (13)	^**^	^**^	^**^	6
Cheng et al. (23)	^**^	^**^	^**^	6
Fishbein et al. (20)	^**^	^**^	^***^	7
Kern et al. (25)	^**^	^**^	^**^	6
Gilaberte et al. (29)	^**^	^***^	^**^	7
Wan et al. (30)	^**^	^***^	^***^	8
Kyung et al. (28)	^**^	^**^	^**^	6
Jackson–Cowan et al. (24)	^**^	^***^	^**^	7
Theodosiou et al. (16)	^**^	^**^	^***^	7
Silverberg et al. (15)	^**^	^***^	^***^	8
McKenzie and Silverberg (31)	^**^	^**^	^***^	7
Lee and Shin (32)	^**^	^***^	^**^	7
Strom et al. (36)	^**^	^***^	^***^	8
Kim et al. (17)	^**^	^***^	^**^	7
Yu and Silverberg (10)	^**^	^***^	^***^	8

### 3.4 Association between AD and mental disorders in adults

**Figure 2:**
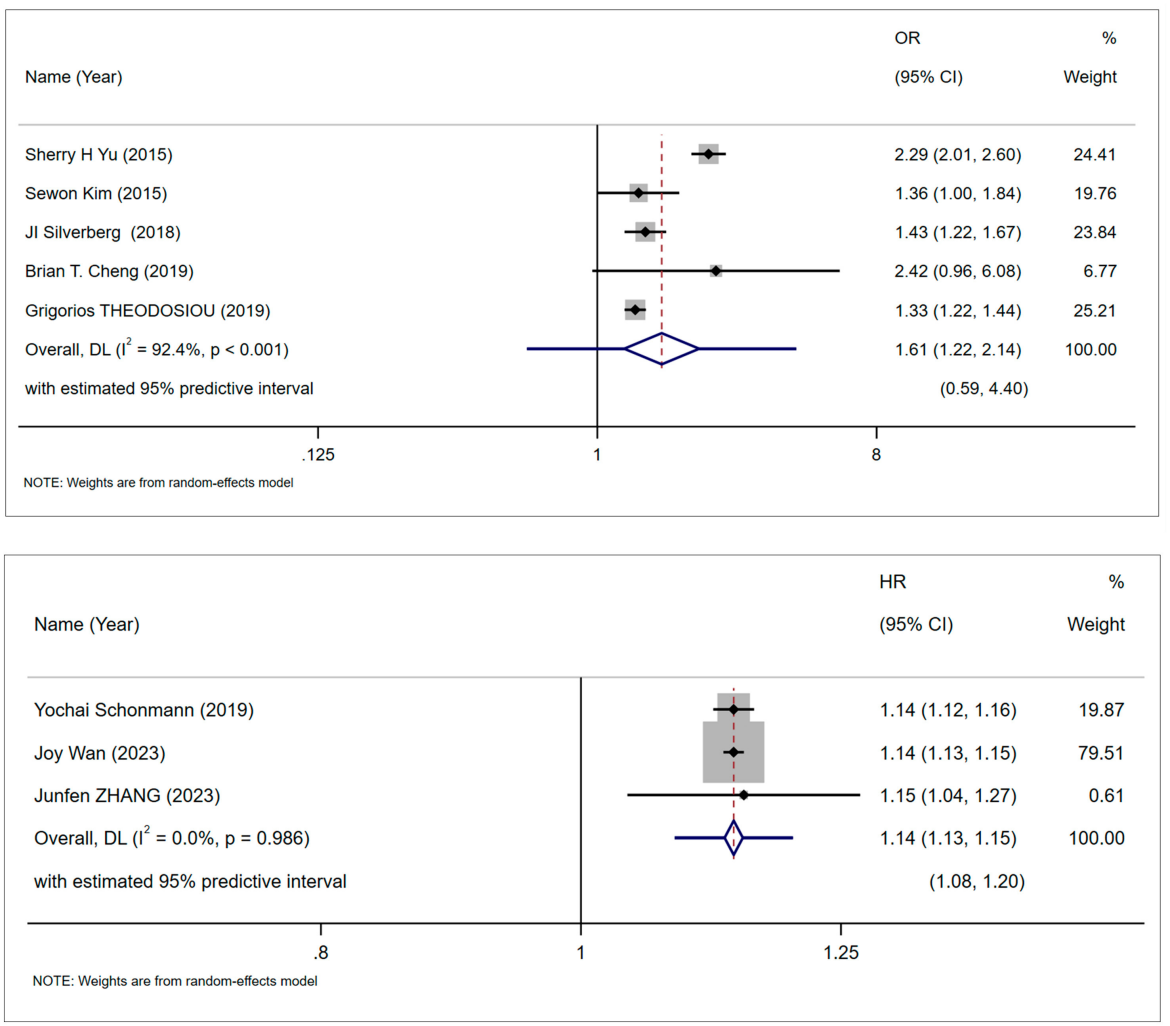
Forest plot. Patients with atopic dermatitis have a significantly increased risk of depression in adults.

**Figure 3:**
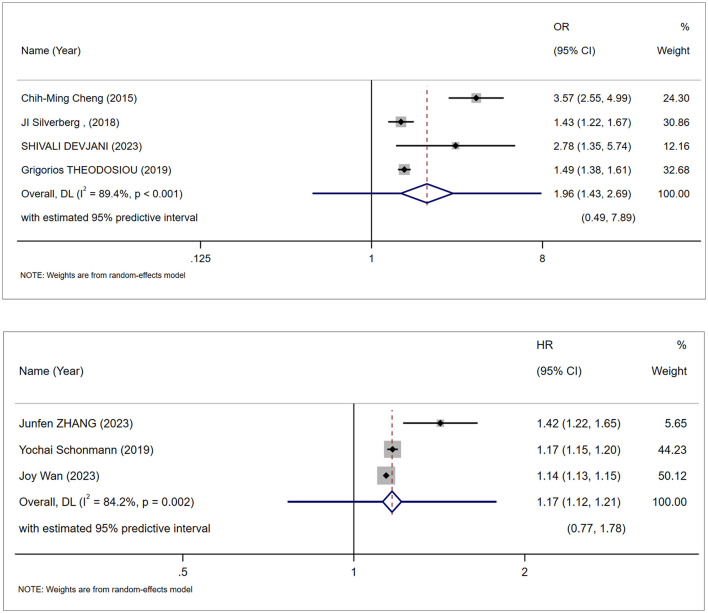
Forest plot. Patients with atopic dermatitis have a significantly increased risk of anxiety in adults.

Among adults with AD, the pooled estimates showed statistically significant positive associations with depression and anxiety, although high heterogeneity was observed for both outcomes (*I*^2^ > 84%). For depression, the pooled OR from cross-sectional studies was 1.61 (95% CI: 1.22–2.14; *I*^2^ = 92.4%, *P* < 0.001), and the pooled HR from cohort studies (plus one case-control study) was 1.14 (95% CI: 1.13–1.15; *I*^2^ = 0.0%, *P* < 0.001) (Figure 2). For anxiety, the pooled OR from cross-sectional studies was 1.96 (95% CI: 1.43–2.69; *I*^2^ = 89.4%, *P* < 0.001), and the pooled HR from cohort studies was 1.17 (95% CI: 1.12–1.21; *I*^2^ = 84.2%, *P* < 0.001) (Figure 3).

Additionally, AD was associated with panic (HR = 1.43, 95% CI: 1.22–1.68; *I*^2^ = 0.0%, *P* < 0.001; Supplementary Figure 1), fatigue (HR = 2.31, 95% CI: 1.07–4.98; *I*^2^ = 66.3%, *P* < 0.001; Supplementary Figure 2), sleep impairment (HR = 1.49, 95% CI: 1.13–1.96; *I*^2^ = 88.7%, *P* < 0.001; Supplementary Figure 3), ADHD (HR = 1.67, 95% CI: 1.08–2.59; *I*^2^ = 95.2%, *P* = 0.020; Supplementary Figure 4), and suicide attempts (HR = 1.31, 95% CI: 1.02–1.68; *I*^2^ = 95.3%, *P*=0.031; Supplementary Figure 5).

In contrast, no statistically significant associations were observed for bipolar disorder (HR = 1.25, 95% CI: 0.91–1.73; *I*^2^ = 58.8%, *P* = 0.176; Supplementary Figure 6), schizophrenia (HR = 1.01, 95% CI: 0.90–1.13; *I*^2^ = 0.0%, *P* = 0.850; Supplementary Figure 7), or eating disorder (HR = 2.11, 95% CI: 0.81–5.52; *I*^2^ = 88.6%, *P* = 0.126; Supplementary Figure 8). These results are summarized in Table 3.

**Table 3:** AD and the risk of mental disorders in adults.

Outcome	Studies (*n*)	Effect size	Pooled effect (95% CI)	*I^2^*	*P*	95%PI
**Depression**	5	OR	1.61 (1.22–2.14)	92.4%	< 0.001	0.59–4.40
	3	HR	1.14 (1.13–1.15)	0.0%	< 0.001	1.08–1.20
**Anxiety**	4	OR	1.96 (1.43–2.69)	89.4%	< 0.001	0.49–7.89
	3	HR	1.17 (1.12–1.21)	84.2%	< 0.001	0.77–1.78
**Bipolar disorder**	2	HR	1.25 (0.91–1.73)	58.8%	0.176	/
**ADHD**	4	HR	1.67 (1.08–2.59)	95.2%	0.020	0.21–13.24
**Panic**	2	HR	1.43 (1.22–1.68)	0.0%	< 0.001	/
**Schizophrenia**	2	HR	1.01 (0.90–1.13)	0.0%	0.850	/
**Fatigue**	3	HR	2.31 (1.07–4.98)	66.3%	< 0.001	0.00–12894.95
**Sleep impairment**	3	HR	1.49 (1.13–1.96)	88.7%	0.0	0.06–37.41
**Suicide attempts**	3	HR	1.31 (1.02–1.68)	95.3%	0.031	0.08–21.15
**Eating disorder**	2	HR	2.11 (0.81–5.52)	88.6%	0.126	/

CI, confidence interval; HR, Hazard Ratio; PI, Prediction Interval.

For meta-analyses with at least three included studies, we calculated 95% PIs, and the results are shown in Table 3. Among the nine eligible analyses, eight PIs were wider than their corresponding 95% CIs and most crossed the null value of 1.0. For instance, the PIs for depression (OR:0.59–4.40), anxiety (OR:0.49–7.89), and ADHD (HR:0.21–13.24) showed considerable uncertainty. Only the HR analysis for depression (*k* = 3, *I*^2^ = 0%) yielded a relatively narrow PI that did not cross 1.0 (1.08–1.20). Notably, the PI for fatigue was extremely wide (0.00–12,894.95), reflecting the high instability of prediction intervals when based on few studies with moderate heterogeneity.

It is worth noting that several outcomes (ADHD, anxiety, suicide attempts, sleep impairment, and eating disorder) exhibited high heterogeneity (*I*^2^ > 80%), as did the OR analysis for depression. This suggests potential variations across studies in population characteristics, assessment methods, or study design. Caution is warranted when interpreting these findings.

### 3.5 Association between AD and mental disorders in children and adolescents

Among children and adolescents, AD was associated with elevated risk estimates for several neurodevelopmental, emotional, and sleep-related outcomes, although the very high heterogeneity (*I*^2^ > 90% for most outcomes) warrants cautious interpretation.

**Figure 4:**
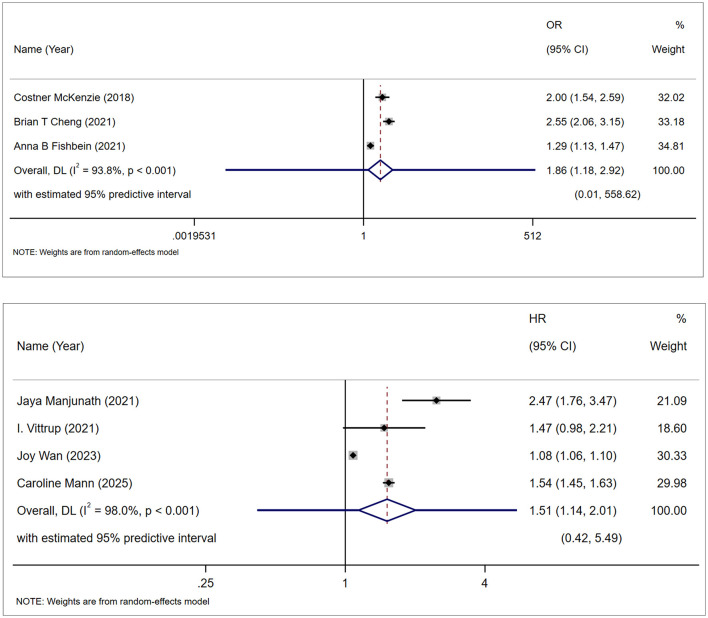
Forest plot. Patients with atopic dermatitis have a significantly increased risk of anxiety in children/adolescents.

For anxiety, cohort/case-control studies yielded a pooled HR of 1.51 (95% CI: 1.14–2.01; *I*^2^ = 98.0%, *P* < 0.001), whereas cross-sectional studies produced a pooled OR of 1.86 (95% CI: 1.16–2.92; *I*^2^ = 93.8%, *P* < 0.001) (Figure 4).

**Figure 5:**
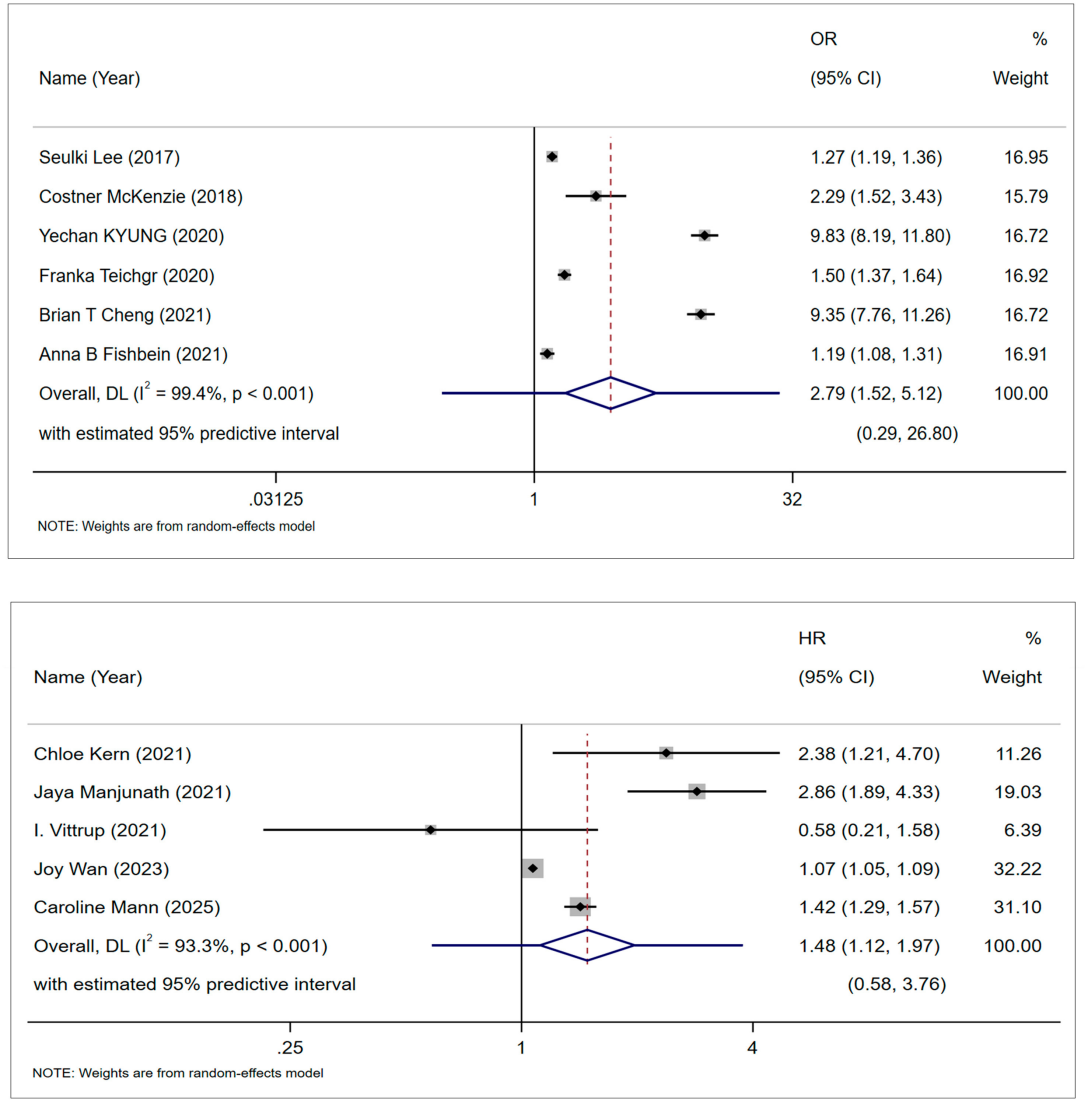
Forest plot. Patients with atopic dermatitis have a significantly increased risk of depression in children/adolescents.

Regarding depression, the pooled OR from cross-sectional studies was 2.79 (95% CI: 1.52–5.12; *I*^2^ = 99.4%, *P* < 0.001), notably higher than the pooled HR from cohort/case-control studies (1.48, 95% CI: 1.12–1.97; *I*^2^ = 93.3%, *P* < 0.001) (Figure 5), a difference that may reflect variations in study design or outcome measurement.

**Figure 6:**
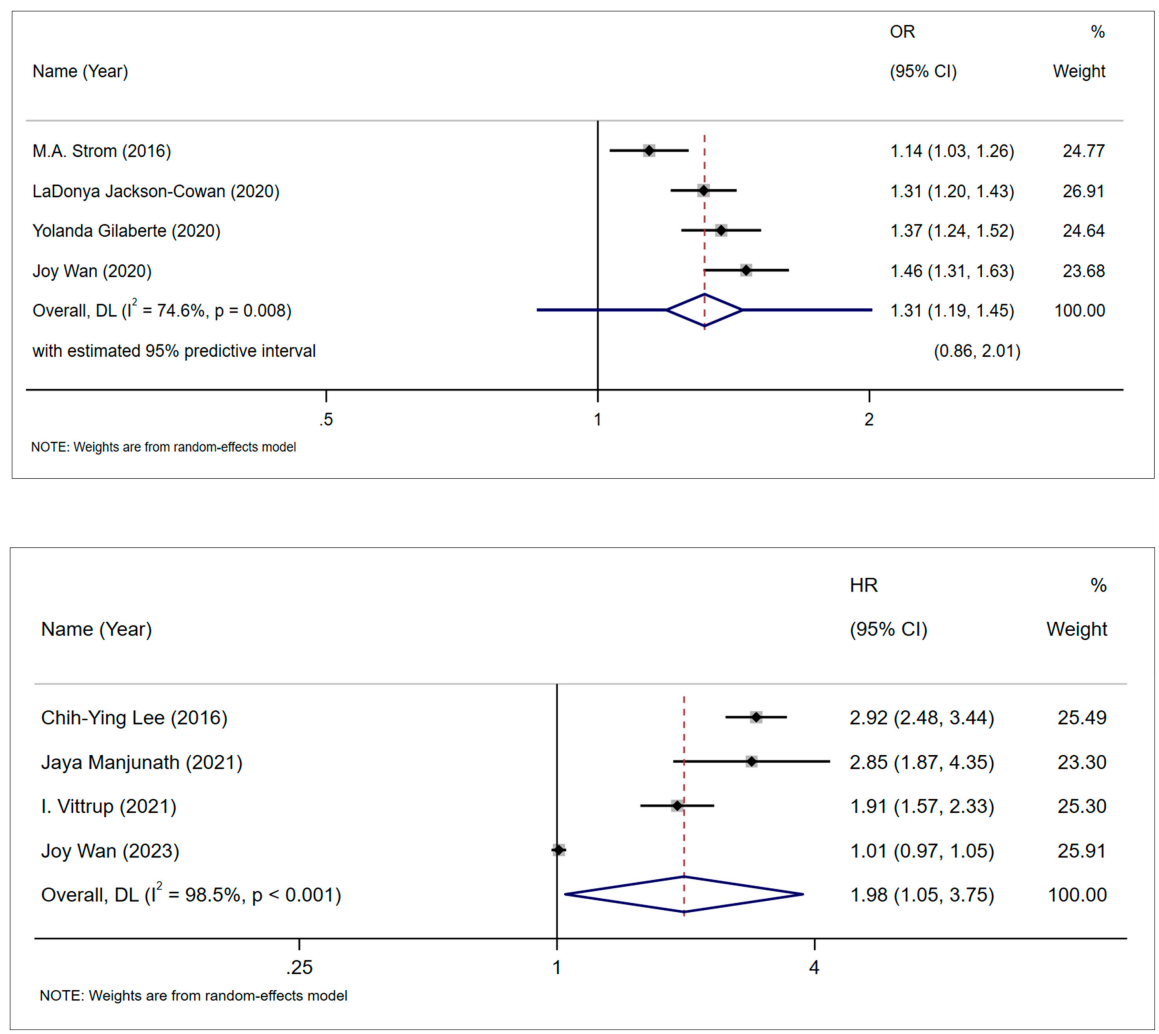
Forest plot. Patients with atopic dermatitis have a significantly increased risk of ADHD in children/adolescents.

For ADHD, cross-sectional studies showed a pooled OR of 1.31 (95% CI: 1.19–1.45; *I*^2^ = 74.6%, *P* < 0.001), and cohort/case-control studies showed a pooled HR of 1.98 (95% CI: 1.05–3.75; *I*^2^ = 98.5%, *P* < 0.001) (Figure 6).

Additionally, AD was associated with autism spectrum disorder (HR = 2.21, 95% CI: 1.15–4.23; *I*^2^ = 96.9%, *P* = 0.017; Supplementary Figure 9), stress (HR = 2.48, 95% CI: 1.75–3.50; *I*^2^ = 80.6%, *P* < 0.001; Supplementary Figure 10), suicide attempts (HR = 2.22, 95% CI: 1.24–3.98; *I*^2^ = 98.9%, *P* = 0.007; Supplementary Figure 11), and sleep impairment (HR = 2.77, 95% CI: 1.30–5.90; *I*^2^ = 92.7%, *P* = 0.008; Supplementary Figure 12).

For meta-analyses with at least three studies, we calculated 95% PIs (Table 4). All PIs crossed 1.0, and most were very wide. The PI for ADHD OR (0.86–2.01) was comparatively narrower than the others, although it still spanned the null. The extremely wide PIs observed for several outcomes (e.g., anxiety, autism, sleep impairment) are consistent with the very high heterogeneity (*I*^2^ > 90%) and small number of studies (*k* = 3), which render these prediction intervals of limited practical value.

**Table 4:** AD and the risk of mental disorders in children/adolescents.

Outcome	Studies (*n*)	Effect size	Pooled effect (95% CI)	*I^2^*	*P*	95%PI
**Depression**	6	OR	2.79 (1.52–5.12)	99.4%	< 0.001	0.29–26.80
	5	HR	1.48 (1.12–1.97)	93.3%	< 0.001	0.58–3.76
**Anxiety**	3	OR	1.86 (1.16–2.92)	93.8%	< 0.001	0.01–558.62
	4	HR	1.51 (1.14–2.01)	98.0%	< 0.001	0.42–5.49
**ADHD**	4	OR	1.31 (1.19–1.45)	74.6%	< 0.001	0.86–2.01
	4	HR	1.98 (1.05–3.75)	98.5%	< 0.001	0.09–43.58
**Autism**	3	HR	2.21 (1.15–4.23)	96.9%	0.017	0.00–7551.66
**Stress**	2	HR	2.48 (1.75–3.50)	80.6%	< 0.001	/
**Sleep impairment**	3	HR	2.77 (1.30–5.90)	92.7%	0.008	0.00–31933.58
**Suicide attempts**	4	HR	2.22 (1.24–3.98)	98.9%	0.007	0.15–32.85

CI, confidence interval; HR, Hazard Ratio; PI, Prediction Interval.

### 3.6 Sensitivity analysis

A leave-one-out sensitivity analysis was performed to assess the robustness of the pooled estimates. Sequentially removing each study and recalculating the summary effect size showed that for all outcomes with more than four studies, the overall effect estimate and its 95% CI did not change substantially, indicating that the findings were not driven by any single study (Supplementary Figures 13–22).

## 4 Discussion

### 4.1 Main findings

Children and adolescents with AD had numerically higher risk estimates for depression, anxiety, and suicidality compared with adults, as well as elevated risks for ADHD and autism. However, these outcomes are conceptually distinct—ranging from internalizing disorders (depression, anxiety) to neurodevelopmental conditions (ADHD, autism) and to sleep impairment and suicidal behavior—and should be interpreted separately rather than as a single “psychological comorbidity” entity.

One possible explanation is that minors are in a critical period of ongoing physical and psychological development and often face multiple health issues simultaneously. Previous research has suggested that vulnerability in children and adolescents might be associated with a combination of genetic, immunological, and social factors, but these mechanisms were not directly examined in our study. For example, it has been hypothesized that the chronic low-grade inflammatory state in AD, characterized by elevated cytokines such as IL-4 and IL-13(38, 39), could potentially influence neurodevelopment, though direct evidence from observational studies remains limited (40). Additionally, psychosocial factors—such as peer rejection, bullying, or social avoidance related to visible skin lesions or fatigue—may contribute to the observed risk estimates. However, given the observational nature of the included studies and the very high heterogeneity, these proposed pathways should be considered speculative and warrant further investigation (41, 42).

Despite the very high heterogeneity, the numerically higher risk estimates in children and adolescents warrant consideration of potential underlying mechanisms, although these remain speculative in the context of the current data. Younger individuals may be particularly vulnerable to the psychosocial consequences of a visible, chronic skin condition because their self-identity and peer relationships are still developing. The cumulative burden of disease-related sleep disruption, school absenteeism, and social stigmatization could disproportionately affect emotional regulation and behavioral development during critical developmental windows (43–45). However, because the included studies did not directly measure these pathways, we cannot determine the extent to which they explain the observed age differences. Future research should prospectively collect data on disease course, psychosocial stressors, and developmental milestones to better understand whether and how age modifies the AD-psychopathology relationship.

The very high heterogeneity (*I*^2^ > 90% for most outcomes) that persisted despite sensitivity and subgroup analyses points to substantial differences in study populations, outcome definitions, and unmeasured confounders. For instance, variations in how depression or anxiety were ascertained (e.g., diagnostic interviews vs. validated screening questionnaires), differences in healthcare systems, and incomplete adjustment for socioeconomic status or comorbidities likely contributed to the wide prediction intervals. Consequently, although the pooled estimates consistently showed elevated risk in the pediatric age group, the magnitude of the risk for any single future study remains highly uncertain – an important caveat for clinicians and guideline developers. Rather than treating the numerical point estimates as fixed values, decision-makers should recognize the broad range of plausible effects (as reflected by the prediction intervals) and prioritize local validation of screening and referral strategies. Future meta-analyses would benefit from individual participant data to explore sources of heterogeneity more thoroughly.

Building on these findings, we propose a tiered management framework that transitions from broad screening to precise referral and ultimately to systemic support, designed to systematically embed the recognition and management of depression, anxiety, ADHD, autism, sleep impairment, and suicidality into standard AD clinical practice.

At the first level, universal screening is integrated into frontline care. We recommend routinely incorporating brief, validated psychological screening tools (e.g., SDQ or RCADS) into follow-up visits for all pediatric and adolescent patients with moderate-to-severe AD (46, 47). For adults, clinicians should be trained to actively elicit and interpret reports of emotional distress, sleep problems, and reduced quality of life, treating these not as secondary concerns but as primary indicators of psychological risk.

Based on our meta-analytic findings and clinical expertise, we propose the following expert consensus-based tiered management framework, which is intended as clinical guidance rather than a direct statistical derivation from the pooled data. The second tier establishes a structured pathway for multidisciplinary collaboration and referral. To ensure timely care, a streamlined cross-disciplinary referral pathway between dermatology and mental health services should be established for high-risk individuals. Identifying these patients requires a multidimensional approach that considers factors beyond clinical severity alone, such as early disease onset (before age 5), an unpredictable fluctuating course, a family history of mental health disorders, and observable social or functional withdrawal (Table 5).

**Table 5:** Clinical considerations for psychological comorbidity management in atopic dermatitis patients.

Dimension	Adult AD patients (≥18 years)	Pediatric & adolescent AD patients (< 18 years)
**Risk focus**	•Highest risk in moderate AD •Prioritize subjective distress (itch, sleep, mood) •Assess impact on work & relationships	•**High–risk features:** early onset (< 5y), fluctuating course, family psychiatric history •Monitor self–esteem, peer relations, school performance •Watch for bullying or school avoidance
**Screening**	•Routine ultra–brief screens •Reliable self–report	•Age–appropriate scales (e.g., RCADS) •Dual–track assessment: child + parent/teacher reports (e.g., SDQ)
**Communication**	•Explain “itch–sleep–mood” cycle •Normalize distress as disease response	•Age–friendly language •Assess family understanding & coping
**Referral path**	•Refer if positive screen, suicidal ideation, or severe impairment •Clear referral path to mental health	•Proactive referral for high–risk features or positive screens •Facilitate doctor–school communication (with consent)
**Ecosystem support**	•Encourage family/group support •Recommend stress–reduction programs	•**Empower families:** education & behavioral skills •**Engage schools:** train staff to reduce stigma & provide support

AD, Atopic Dermatitis; y, year; RCADS, Revised Child Anxiety and Depression Scale; SDQ, Strengths and Difficulties Questionnaire.

This framework is based on the meta-analytic findings and is intended as expert-consensus guidance, not as a direct statistical derivation from the pooled data.

At its broadest level, the framework is designed to provide ecosystem-level support by bridging clinical care with the key settings of daily life. Effective management must reach beyond the clinic into the family and school environments. This involves a two-pronged approach: equipping families with knowledge about AD, emotional regulation strategies, and skills-based training to break the itch-scratch cycle, while simultaneously advocating for the integration of AD-specific needs into school health and psychological support systems. Educating school nurses and teachers within this framework helps reduce stigma and misconceptions, thereby fostering a supportive environment that mitigates psychosocial stressors and promotes holistic recovery.

### 4.2 Implications and limitations

No formal statistical comparison was made between pediatric/adolescent and adult groups; thus, the numerically higher risk estimates in younger patients should be interpreted cautiously and require future confirmation. Moreover, despite subgroup and sensitivity analyses, the very high heterogeneity (often *I*^2^ > 90%) for most pooled estimates limits the certainty of our findings, and age-related numerical differences may be influenced by unexplained between-study variation.

Consequently, no causal inferences can be drawn, and all proposed mechanisms are hypothesis-generating rather than conclusive.

In the pediatric subgroup, most prediction intervals were extremely wide and crossed the null, reflecting limited study numbers (typically 3–5 per analysis) and very high heterogeneity (*I*^2^ often >90%). For outcomes with only three studies, PI were particularly unstable (e.g., anxiety OR: 0.01–558.62), as the t-distribution with one degree of freedom dramatically amplifies uncertainty. Thus, although most pooled effect sizes were statistically significant, the predictive value for any individual future study is minimal. The sole exception was the OR analysis for ADHD (k=4, *I*^2^=74.6%), which yielded a comparatively narrower PI (0.86–2.01), suggesting slightly more stable prediction, though caution is still needed.

Another methodological consideration is the inclusion of different observational designs (cohort, case-control, and cross-sectional). Although we kept HRs and ORs separate and did not pool them across designs, inherent differences in temporal inference, confounding control, and recall bias remain. Consequently, the higher risk estimates from cross-sectional analyses (e.g., depression OR in both age groups) compared with cohort-based HRs may partly reflect design-related bias rather than true risk differences. Readers should therefore interpret cross-design comparisons with caution.

Finally, publication bias was not formally tested, as most analyses included few studies (*k* < 10) and the high heterogeneity (*I*^2^ > 80%) makes funnel plot asymmetry tests unreliable. Nevertheless, we cannot exclude the possibility that unpublished null findings exist, which could affect the magnitude of the pooled estimates.

It should be noted that we used NOS-xs rather than AXIS to assess the quality of cross-sectional studies. The literature shows that no gold standard tool exists for cross-sectional studies, and different tools have different evaluation criteria (48). We chose NOS-xs mainly because its framework is consistent with the original NOS used for cohort and case-control studies, allowing uniform quality classification across mixed study designs. However, no single tool can cover all aspects of quality. Using AXIS might lead to different quality ratings. Therefore, our quality results should be interpreted considering this tool choice.

## 5 Conclusion

Our findings suggest that children and adolescents with atopic dermatitis may have a higher risk of common psychological comorbidities such as depression and anxiety than adults, but the substantial heterogeneity across studies limits the certainty of this conclusion. Overall, these results should be interpreted cautiously. To translate this into practice, we advocate for an integrated “stratified age-precise assessment-stepped care” framework, shifting the focus of management from the skin alone to the integrated wellbeing of the mind and body.

## Data Availability

The original contributions presented in the study are included in the article/supplementary material, further inquiries can be directed to the corresponding author.
