# Supplementary material for: Age differences in psychological comorbidity in atopic dermatitis: a systematic review and meta-analysis
Source: Front Public Health. 2026 Jul 7;14:1818252. doi: 10.3389/fpubh.2026.1818252 (PMC13384835; doi:10.3389/fpubh.2026.1818252)
Supplement: Supplementary file 1 [file Supplementary_file_1.docx]

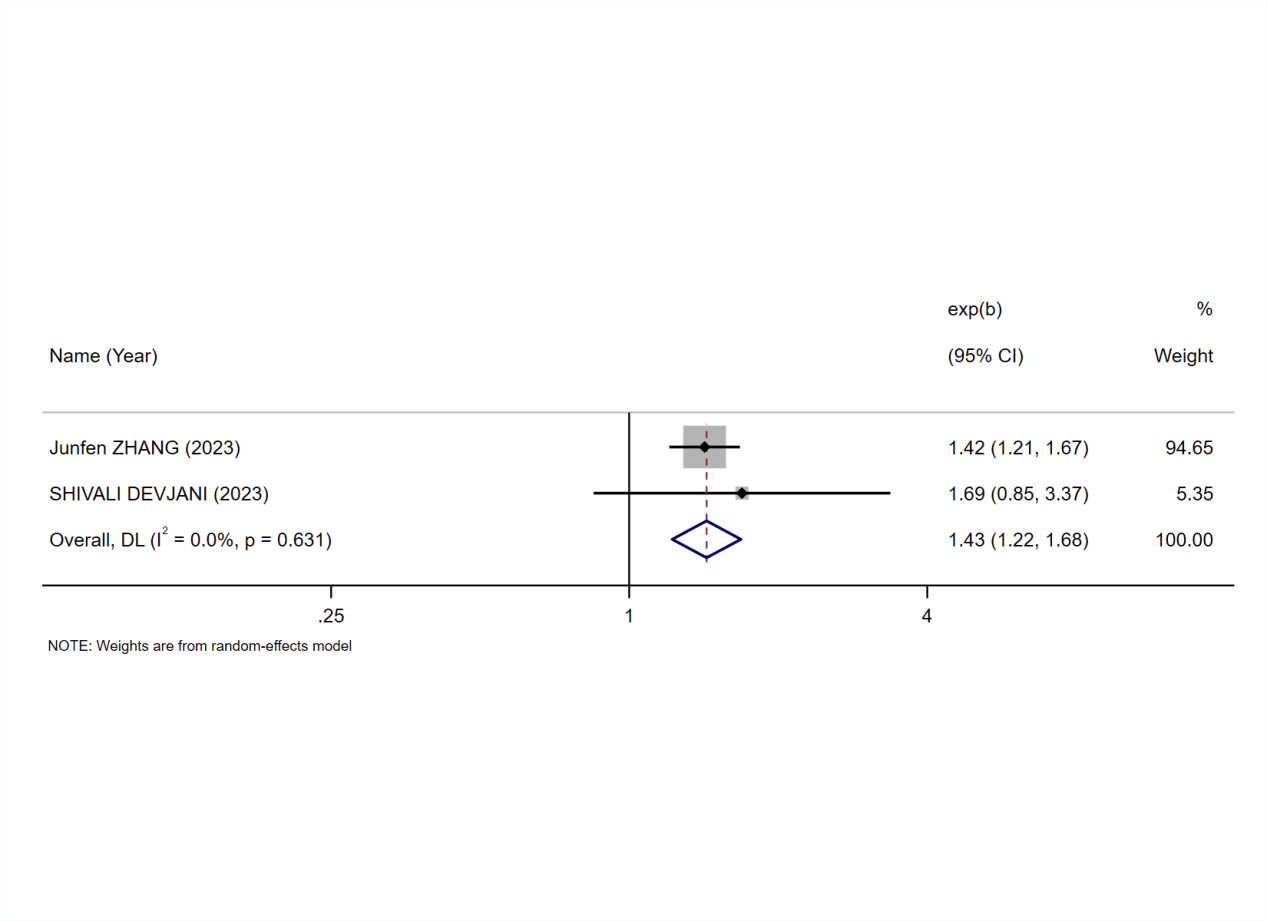


Supplementary Figure 1. Association between AD and panic in adults.


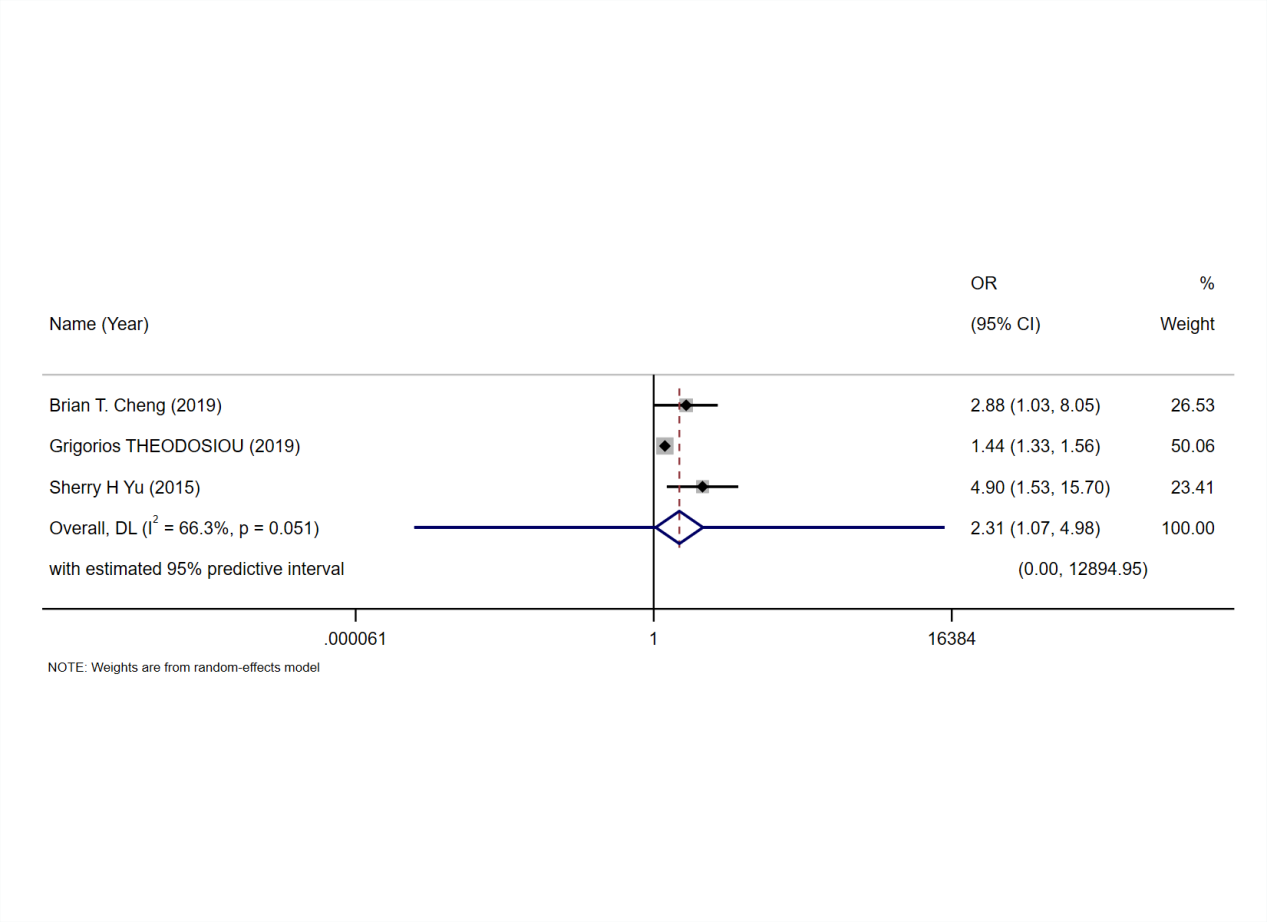


Supplementary Figure 2. Association between AD and fatigue in adults.


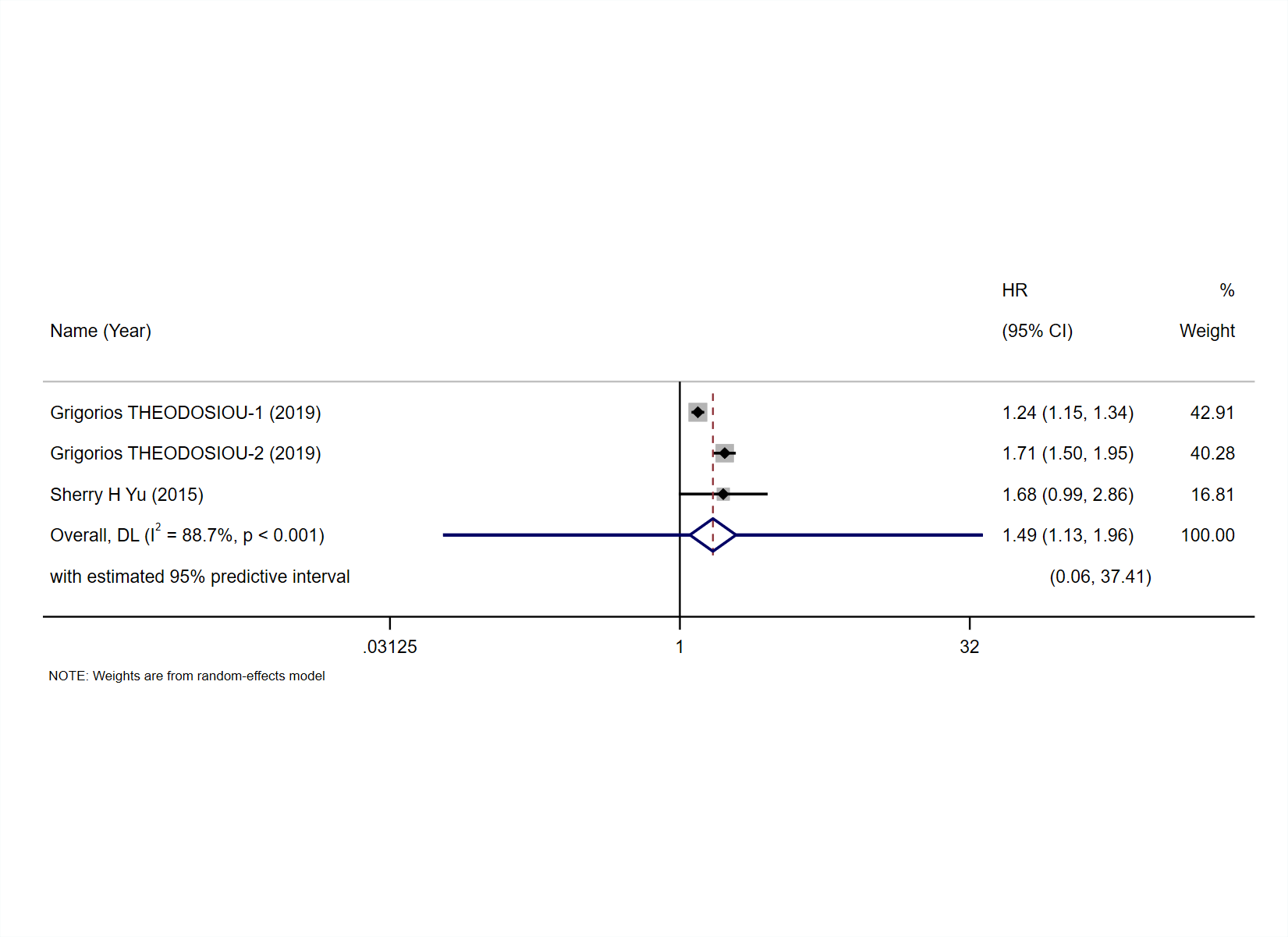


Supplementary Figure 3. Association between AD and sleep impairment in adults.


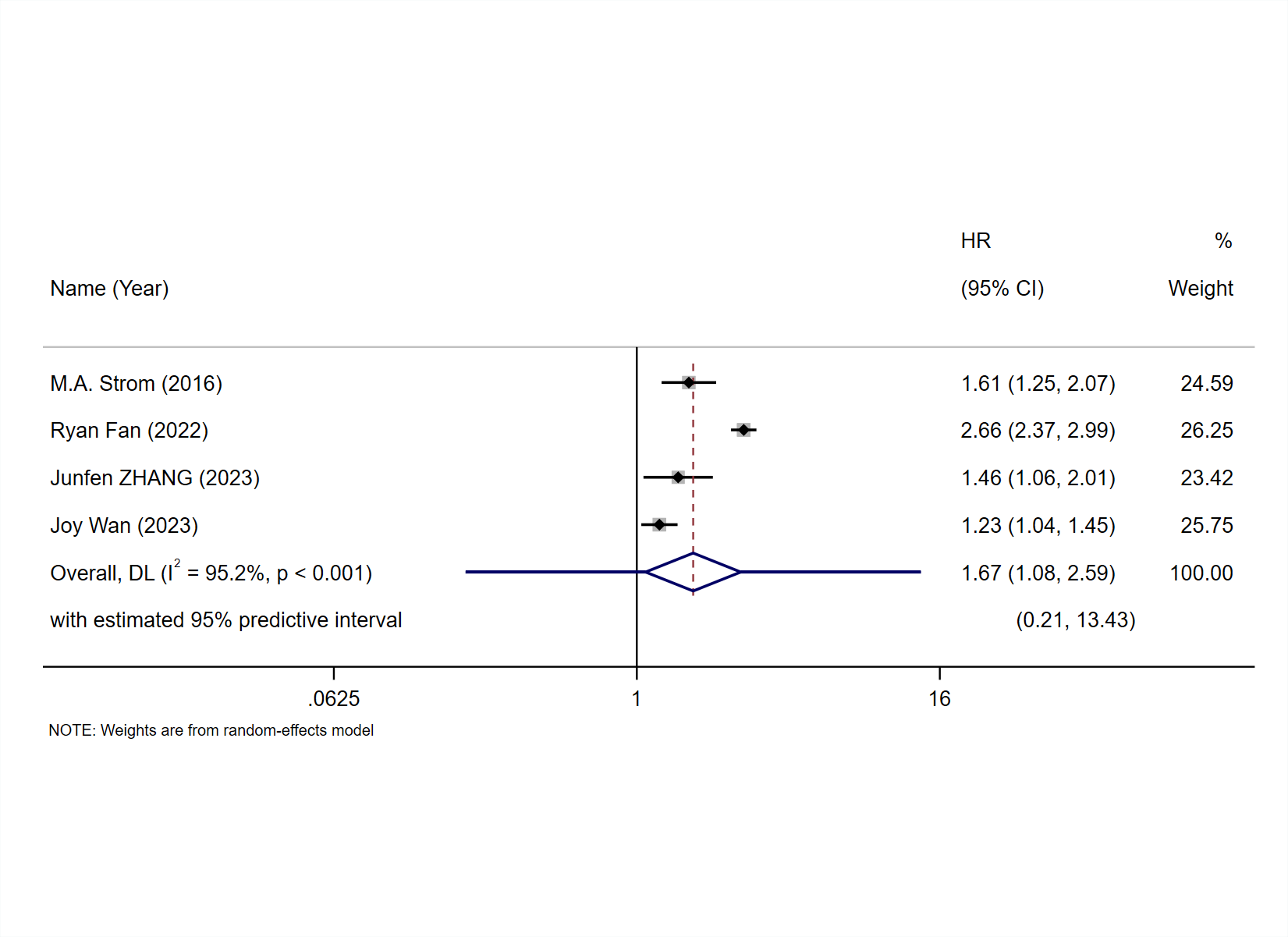


Supplementary Figure 4. Association between AD and attention deficit hyperactivity disorder in adults.


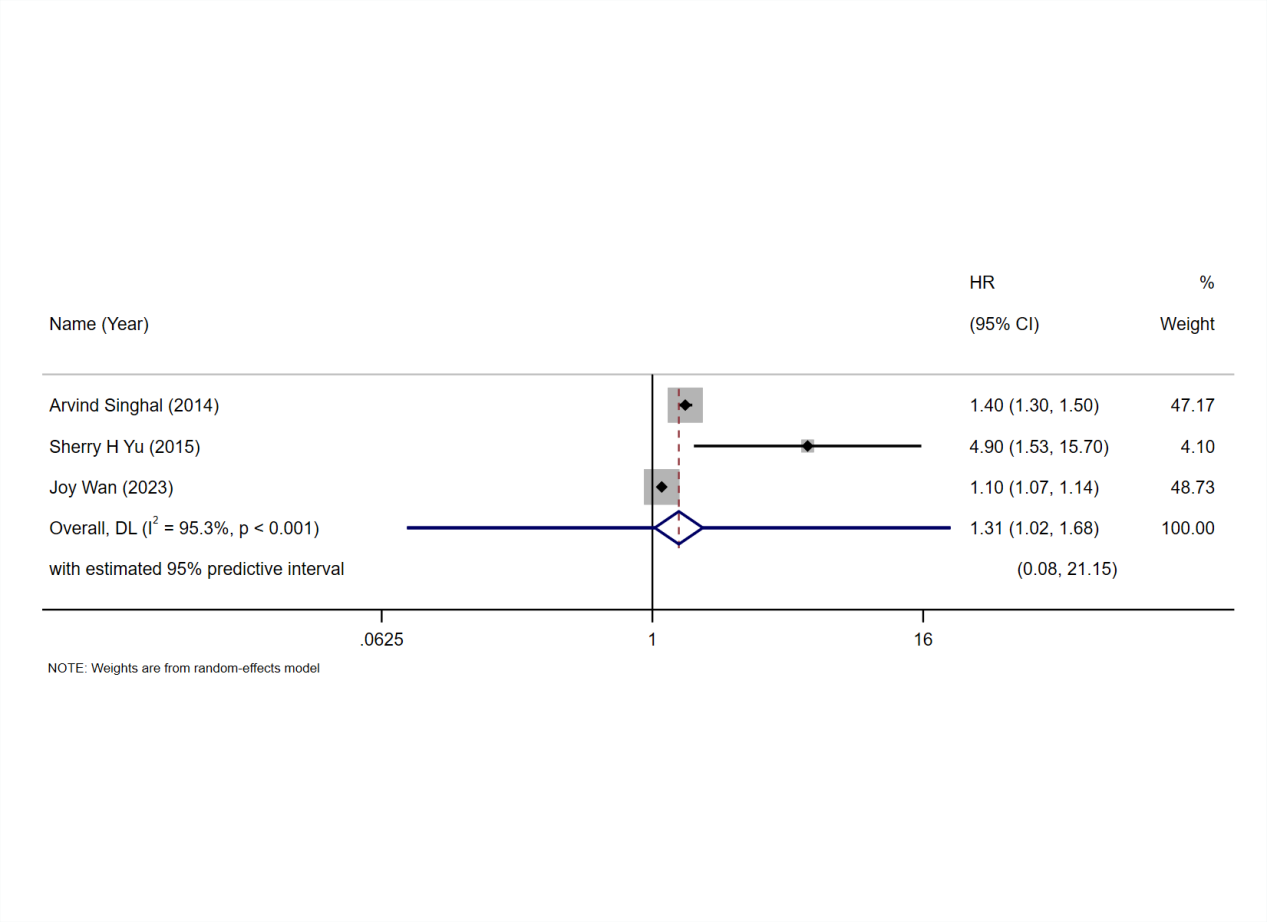


Supplementary Figure 5. Association between AD and suicide attempts in adults.


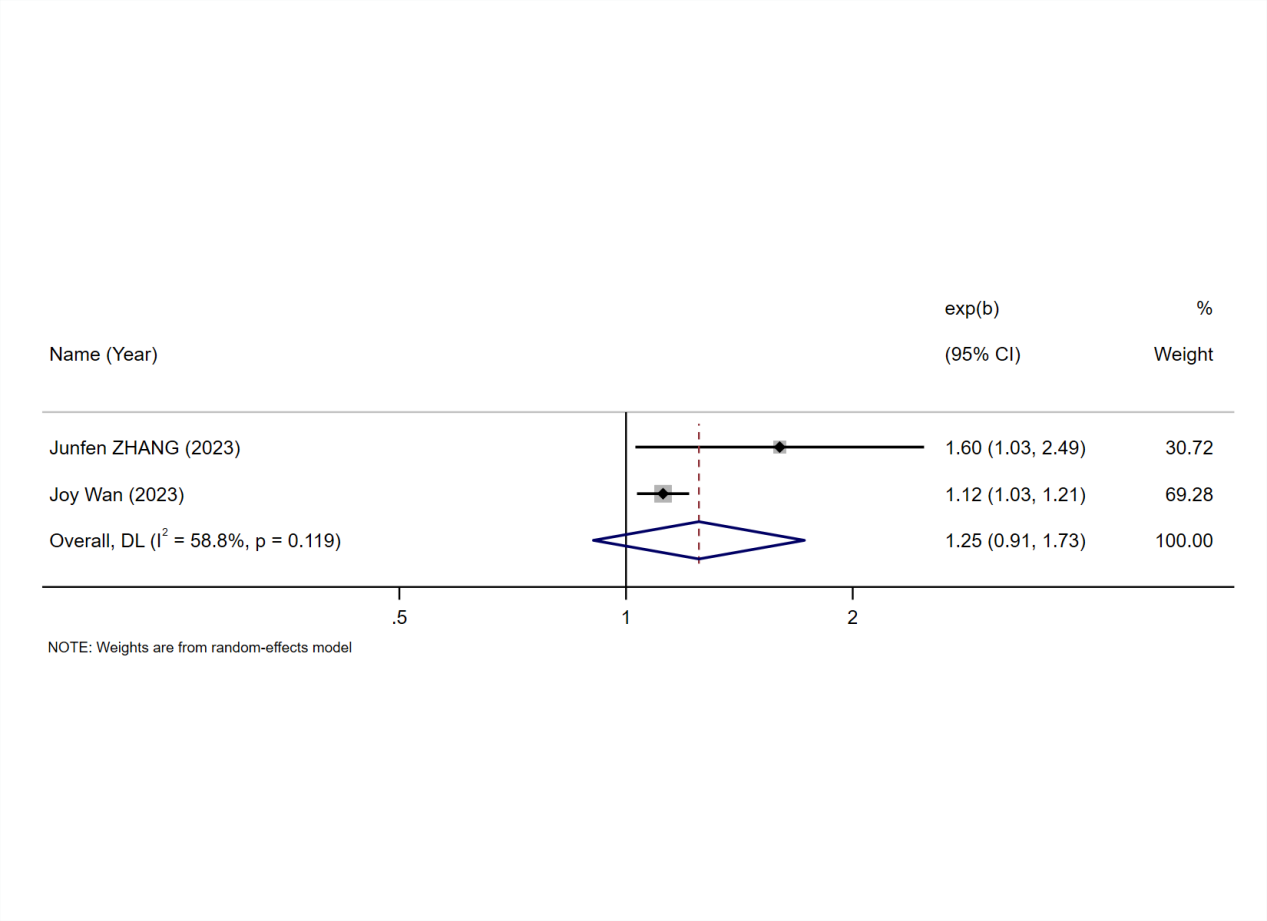


Supplementary Figure 6. Association between AD and bipolar disorder in adults.


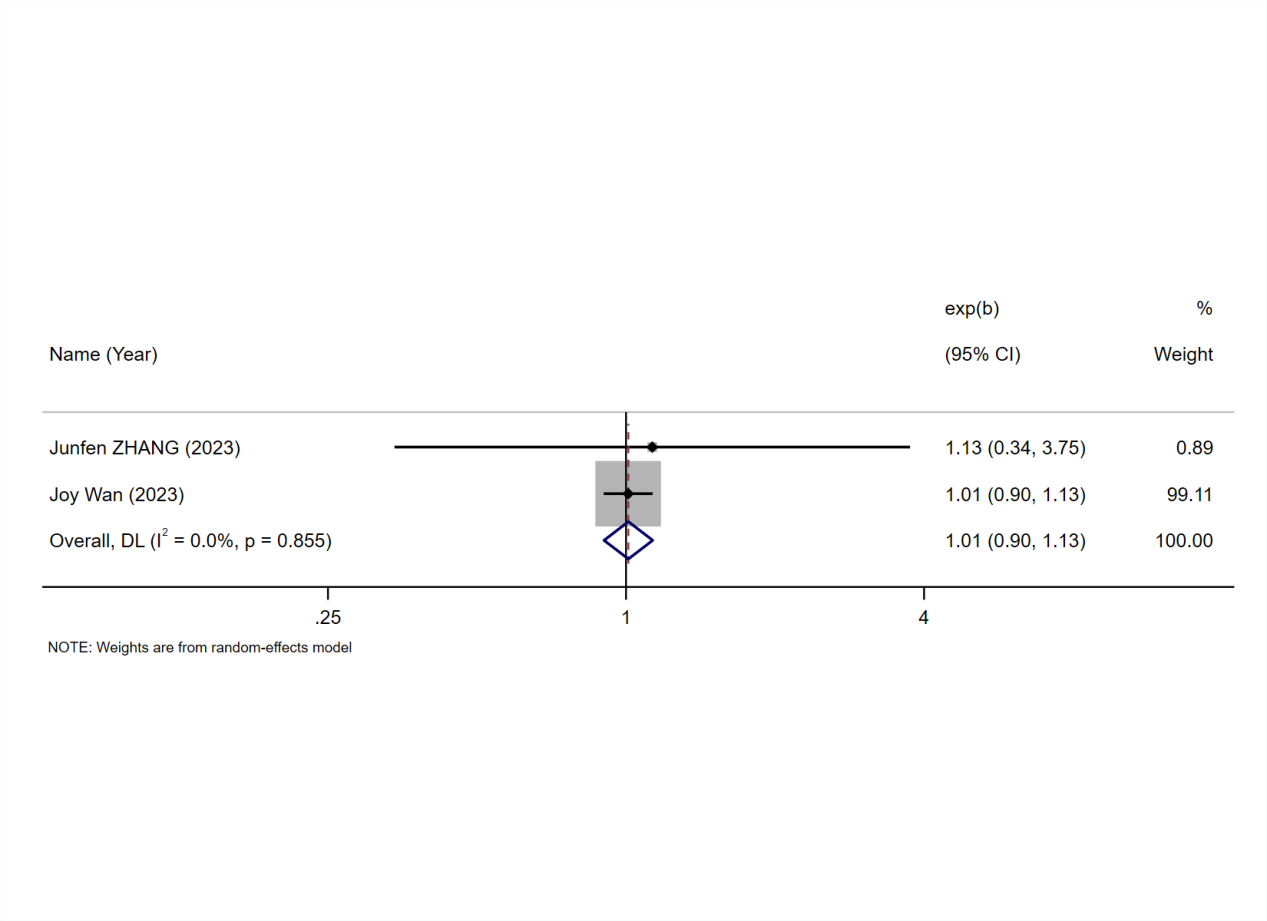


Supplementary Figure 7. Association between AD and schizophrenia in adults.


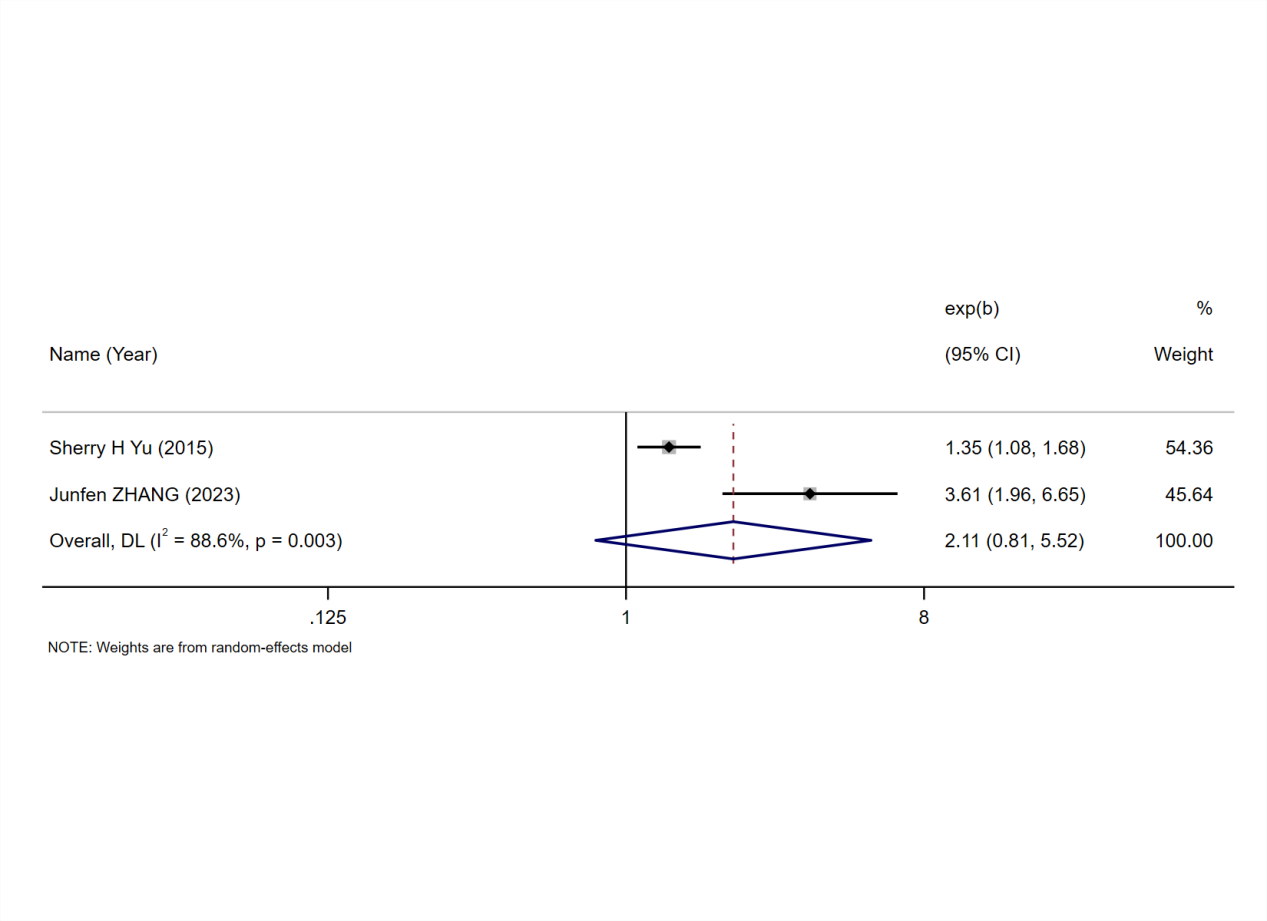


Supplementary Figure 8. Association between AD and eating disorder in adults.


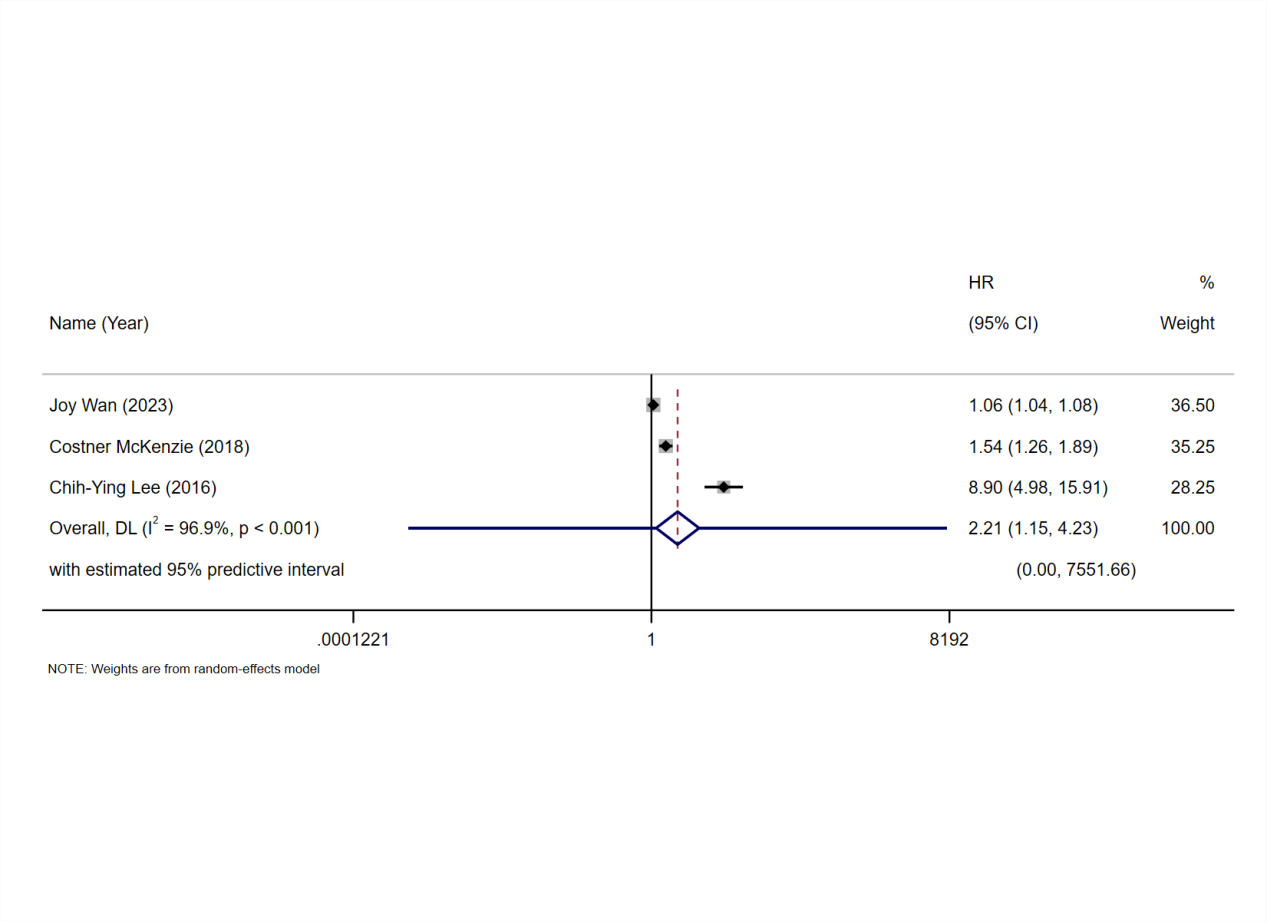


Supplementary Figure 9. Association between AD and autism in children and adolescents


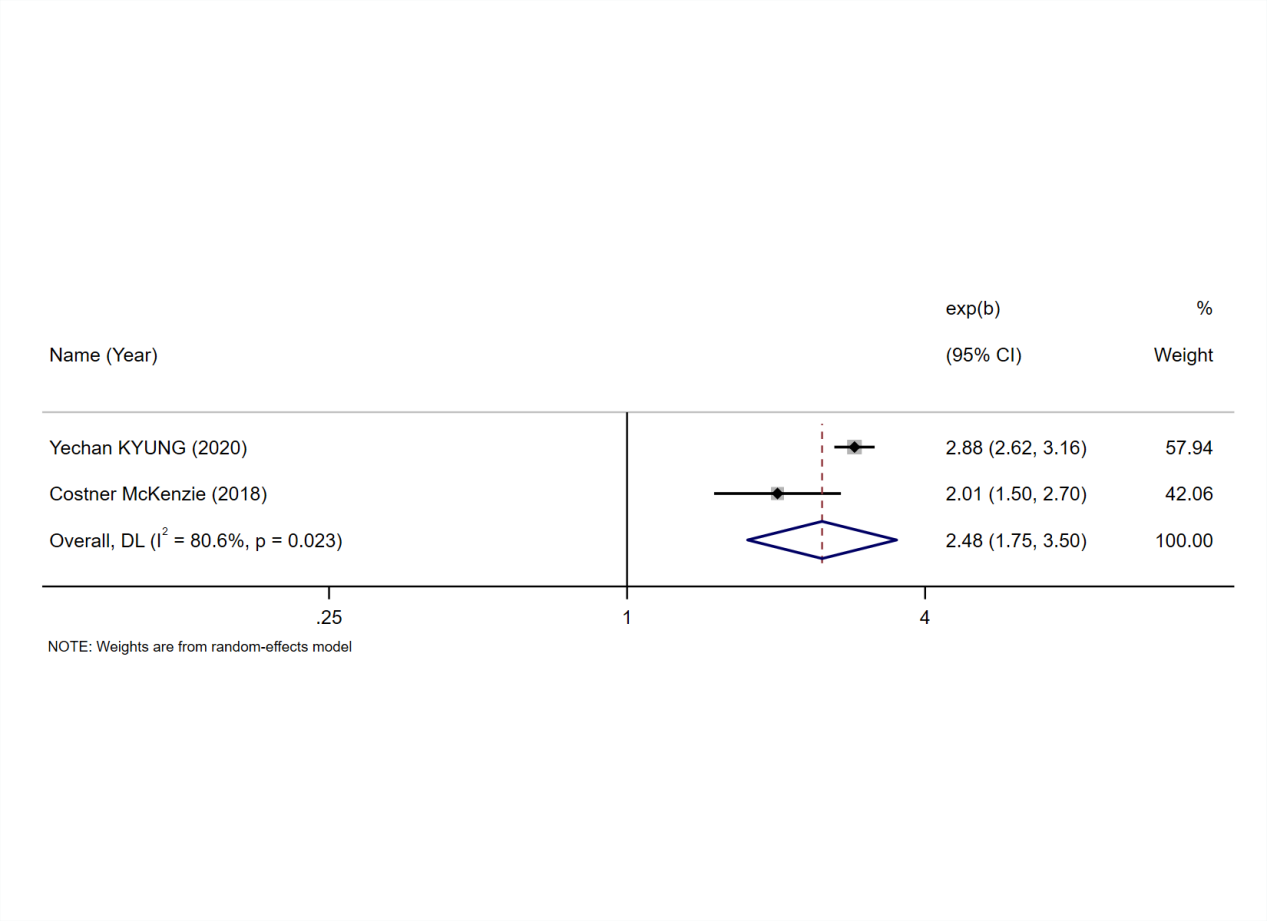


Supplementary Figure 10. Association between AD and stress in children and adolescents


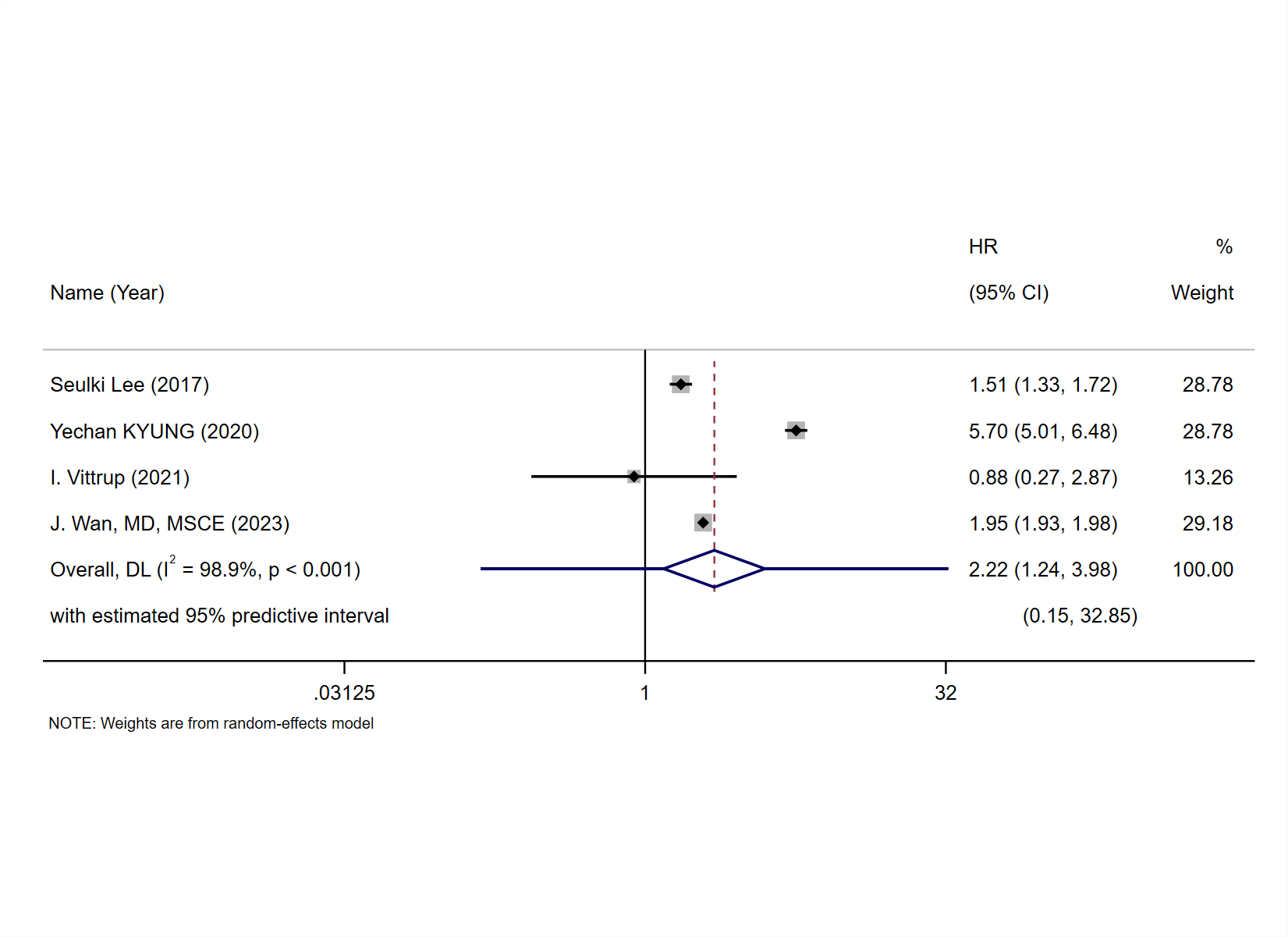


Supplementary Figure 11. Association between AD and suicide attempts in children and adolescents


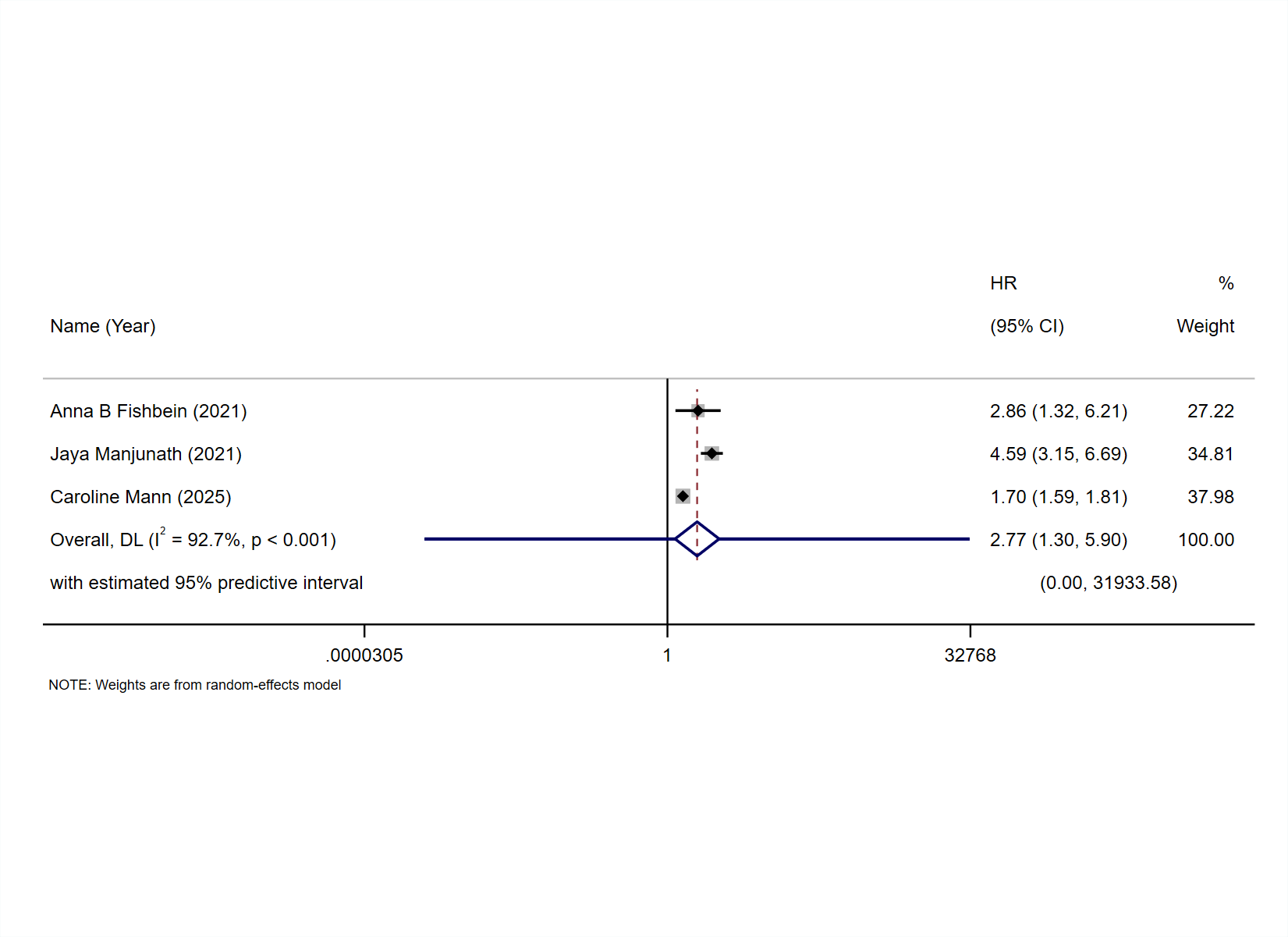


Supplementary Figure 12. Association between AD and sleep impairmentts in children and adolescents


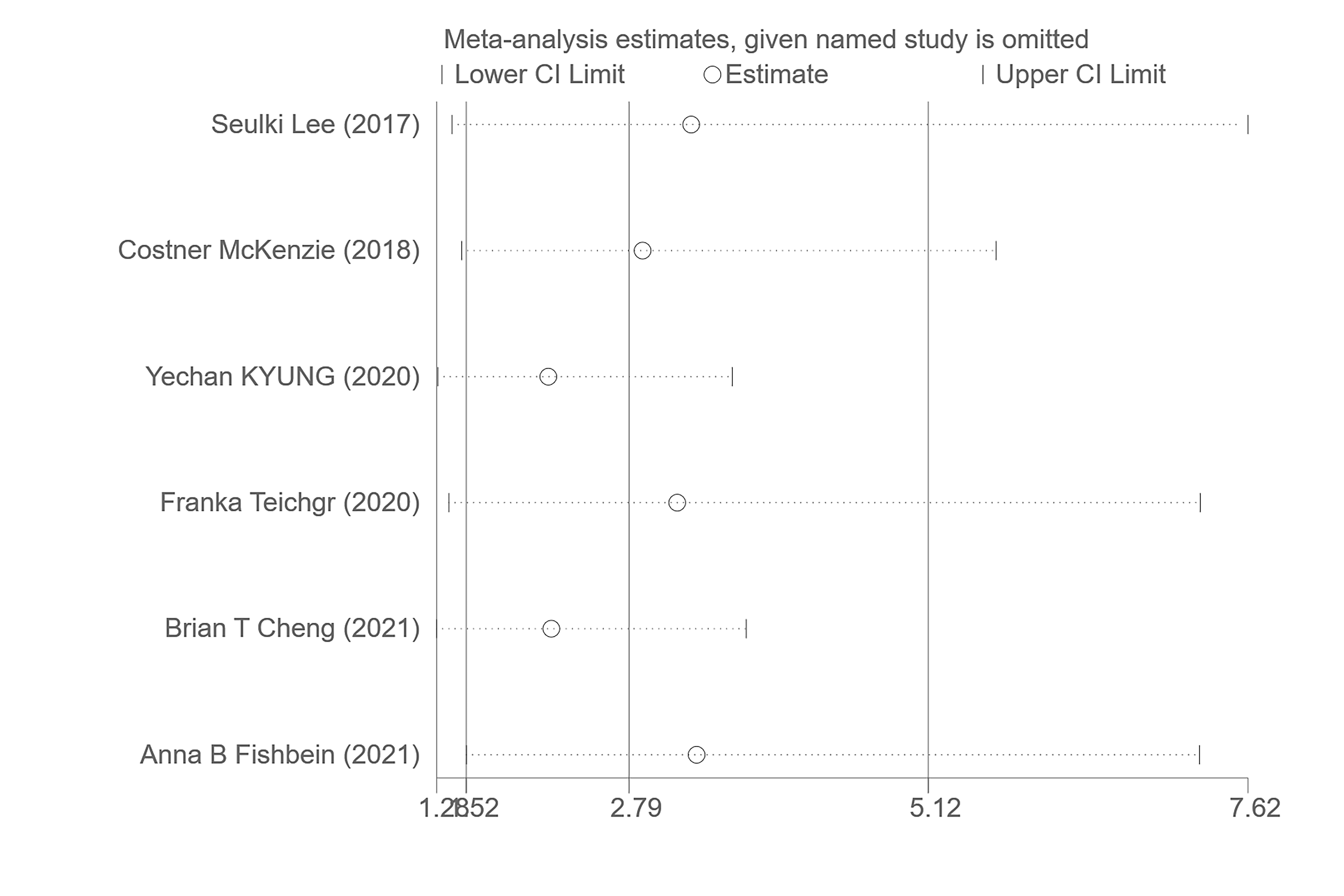


Supplementary Figure 13. Sensitivity analysis for the meta-analysis of depression in children and adolescents(OR).


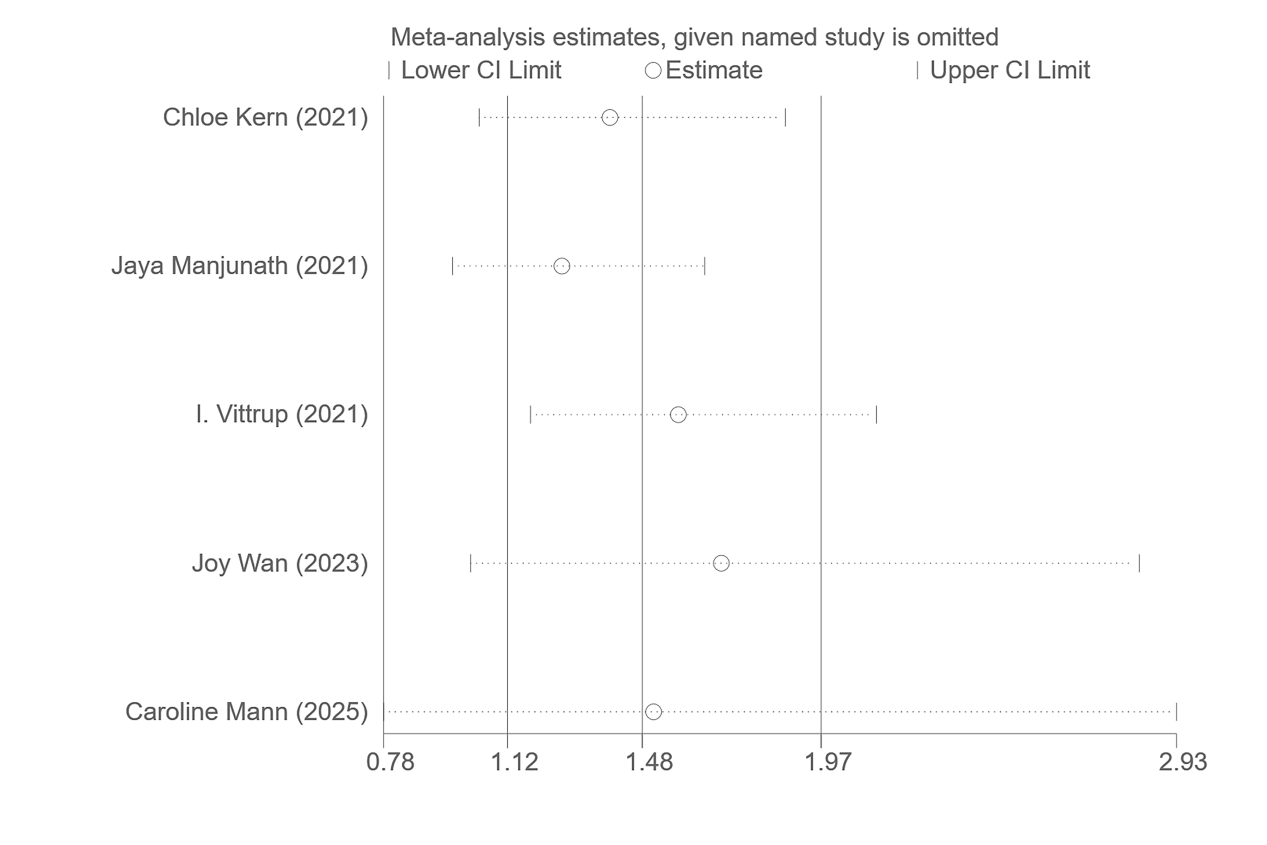


Supplementary Figure 14. Sensitivity analysis for the meta-analysis of depression in children and adolescents(HR).


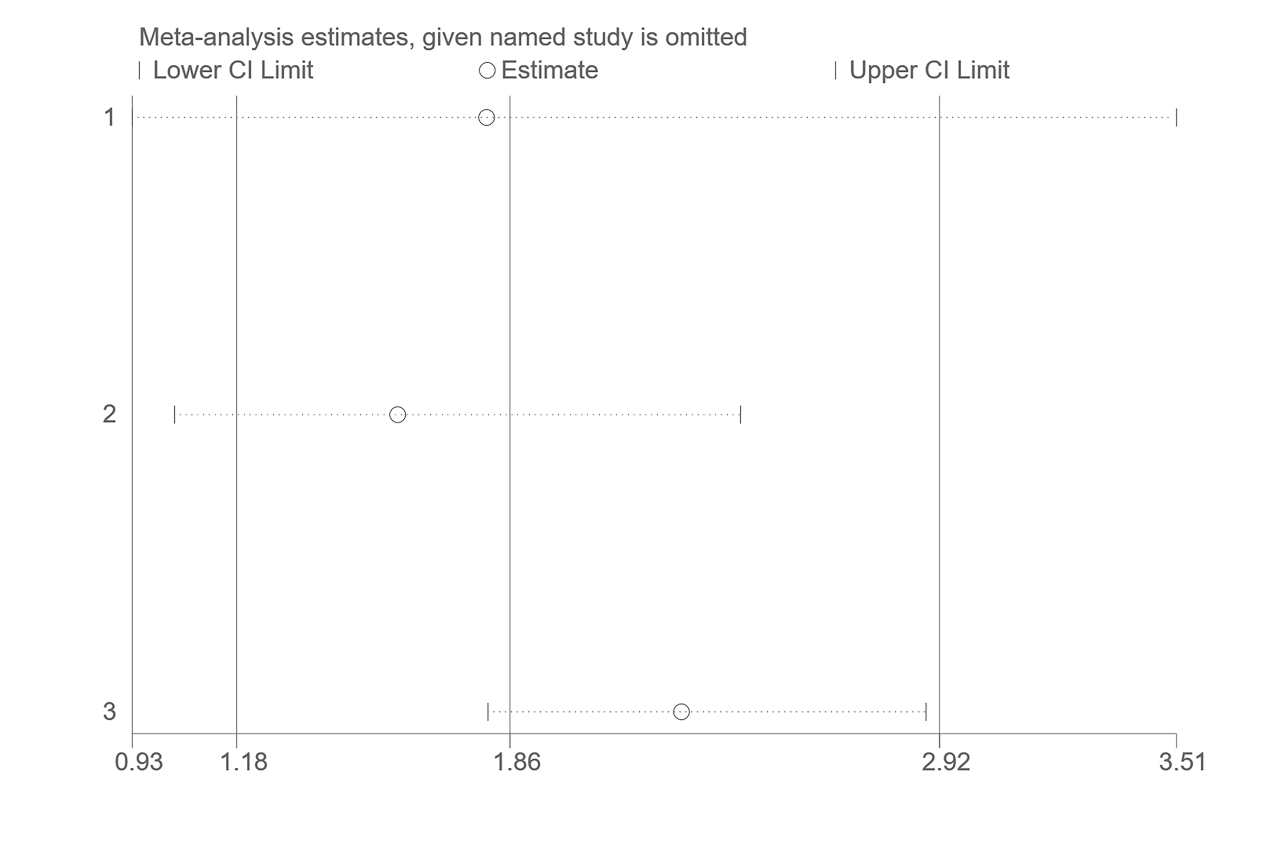


Supplementary Figure 15. Sensitivity analysis for the meta-analysis of anxiety in children and adolescents(OR).


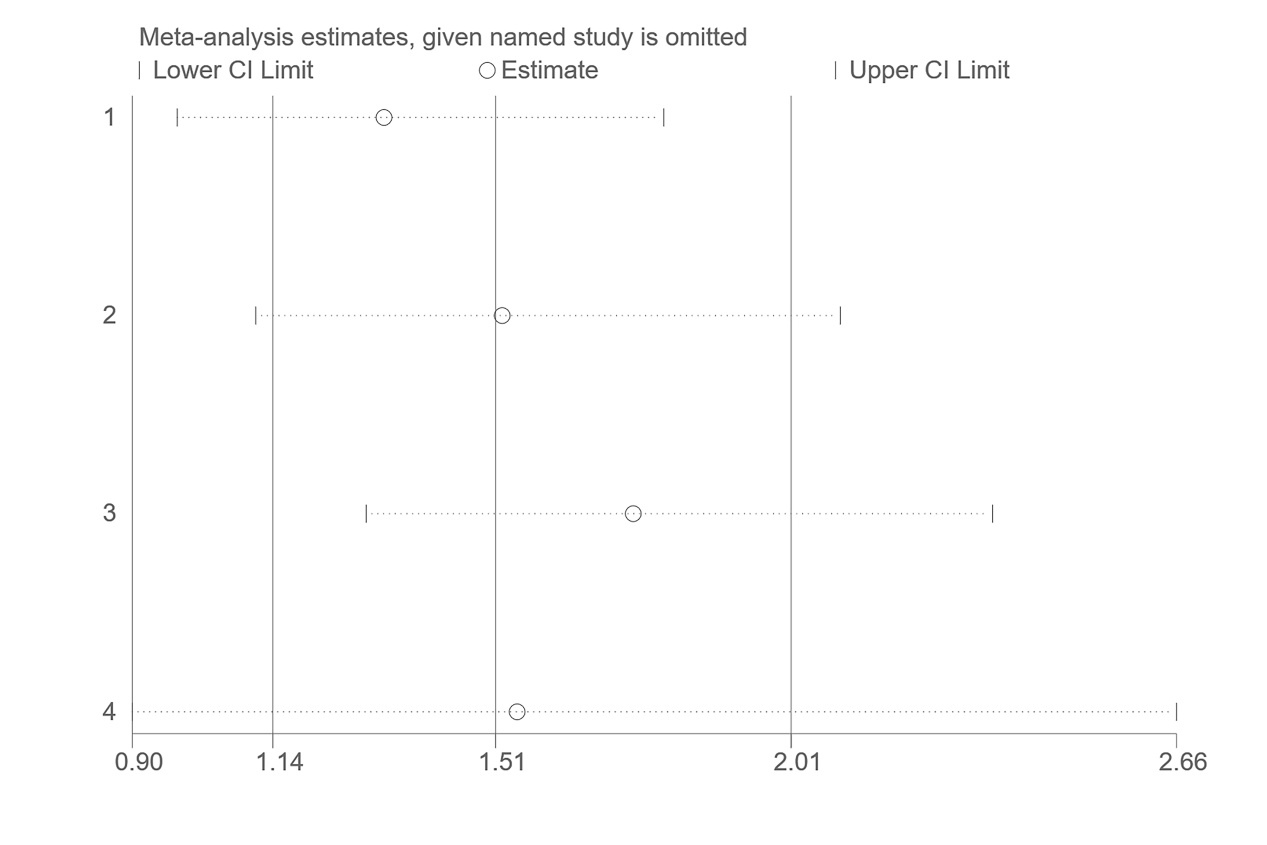


Supplementary Figure 16. Sensitivity analysis for the meta-analysis of anxiety in children and adolescents(HR).


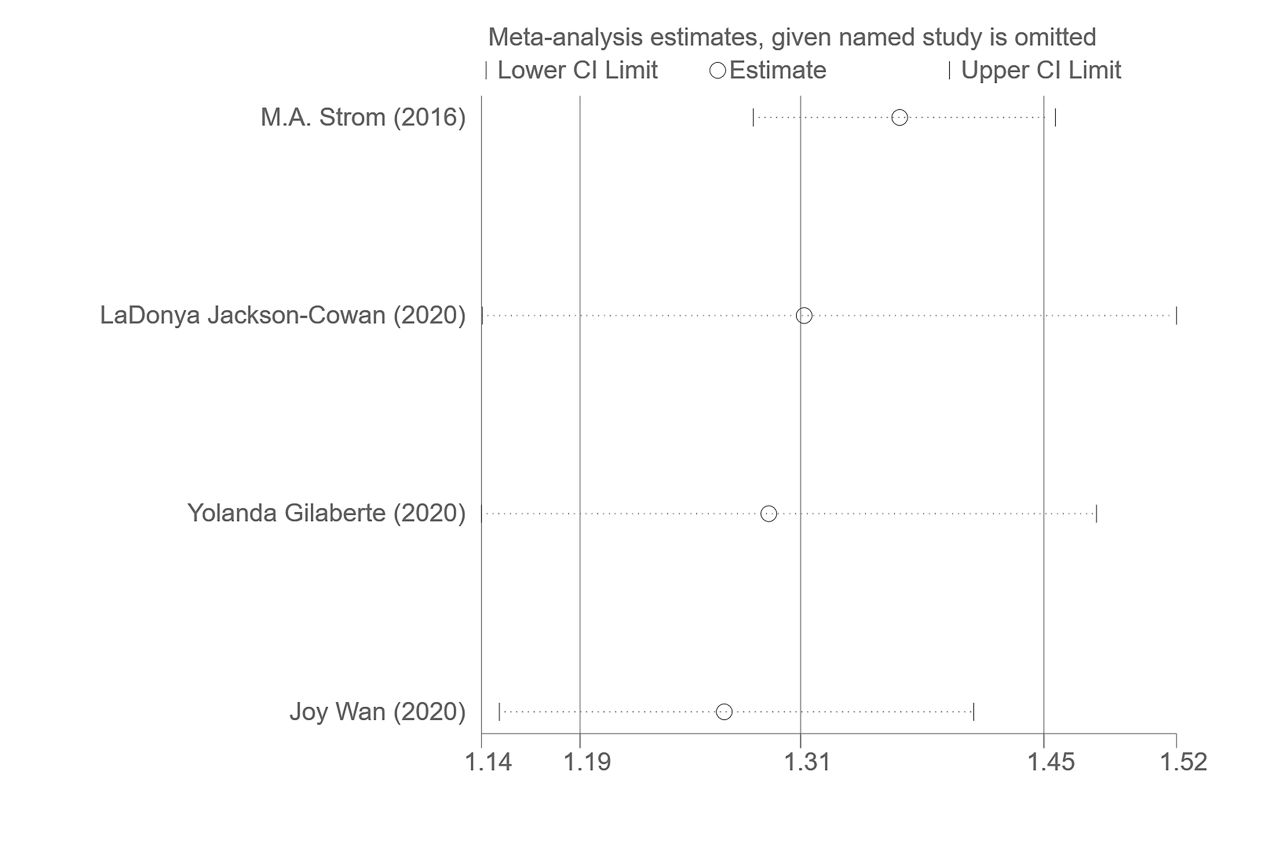


Supplementary Figure 17. Sensitivity analysis for the meta-analysis of ADHD in children and adolescents(OR).


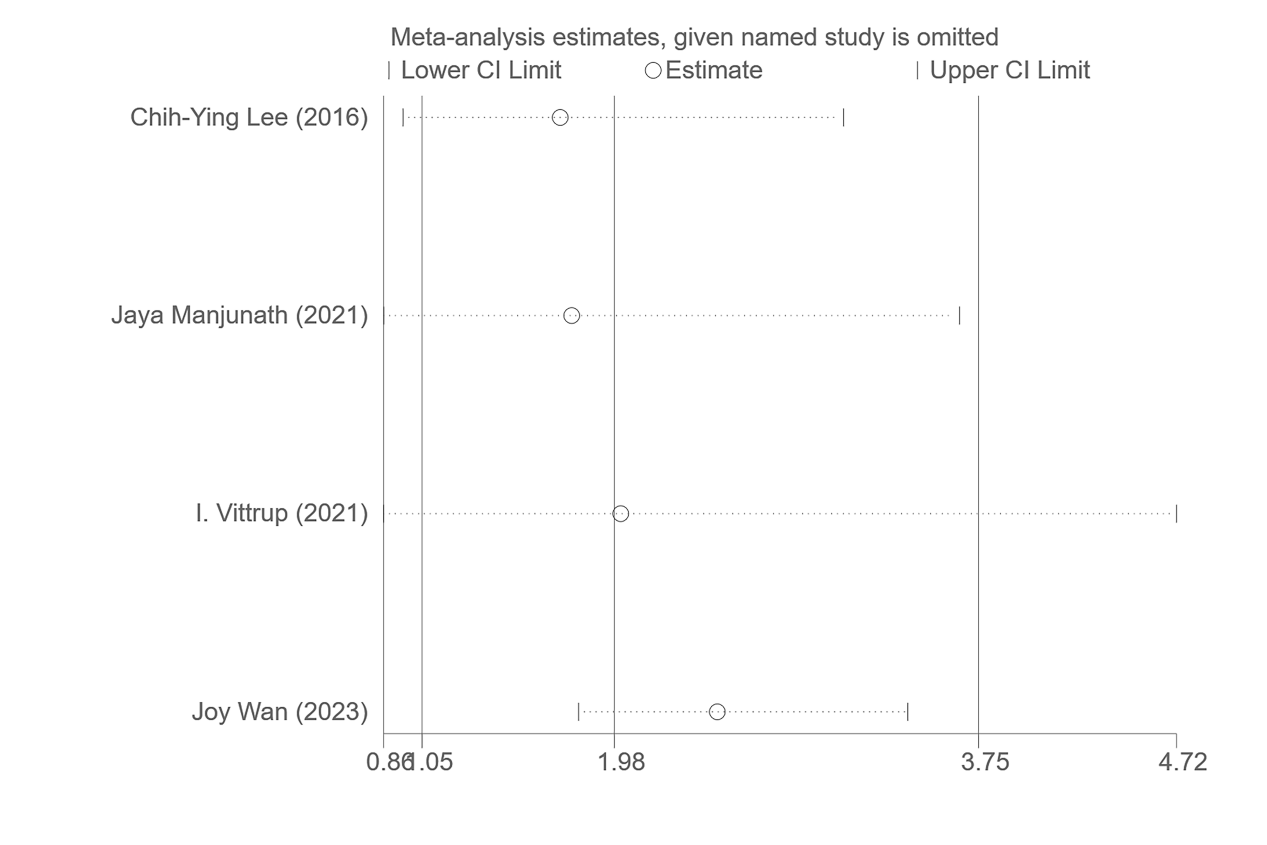


Supplementary Figure 18. Sensitivity analysis for the meta-analysis of ADHD in children and adolescents(HR).


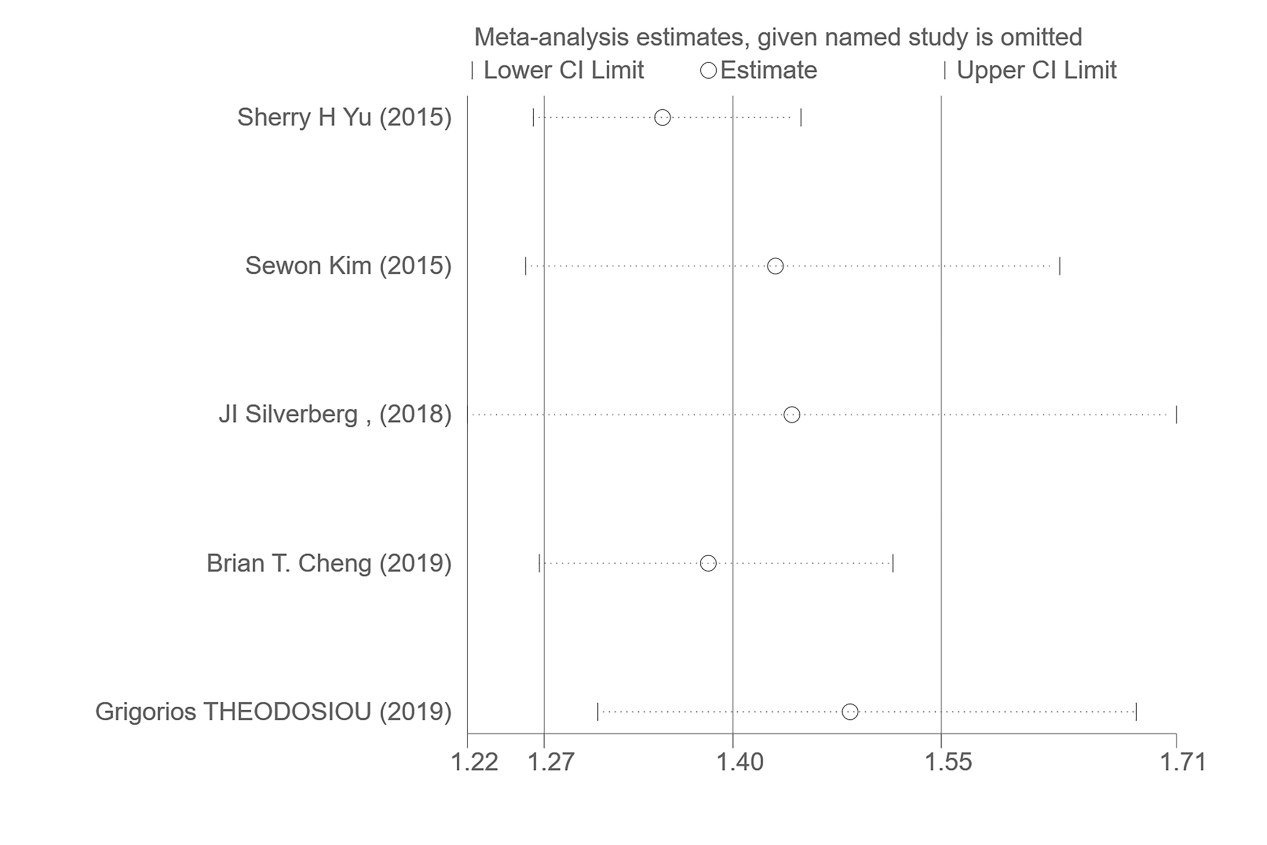


Supplementary Figure 19. Sensitivity analysis for the meta-analysis of depression in adults(OR).


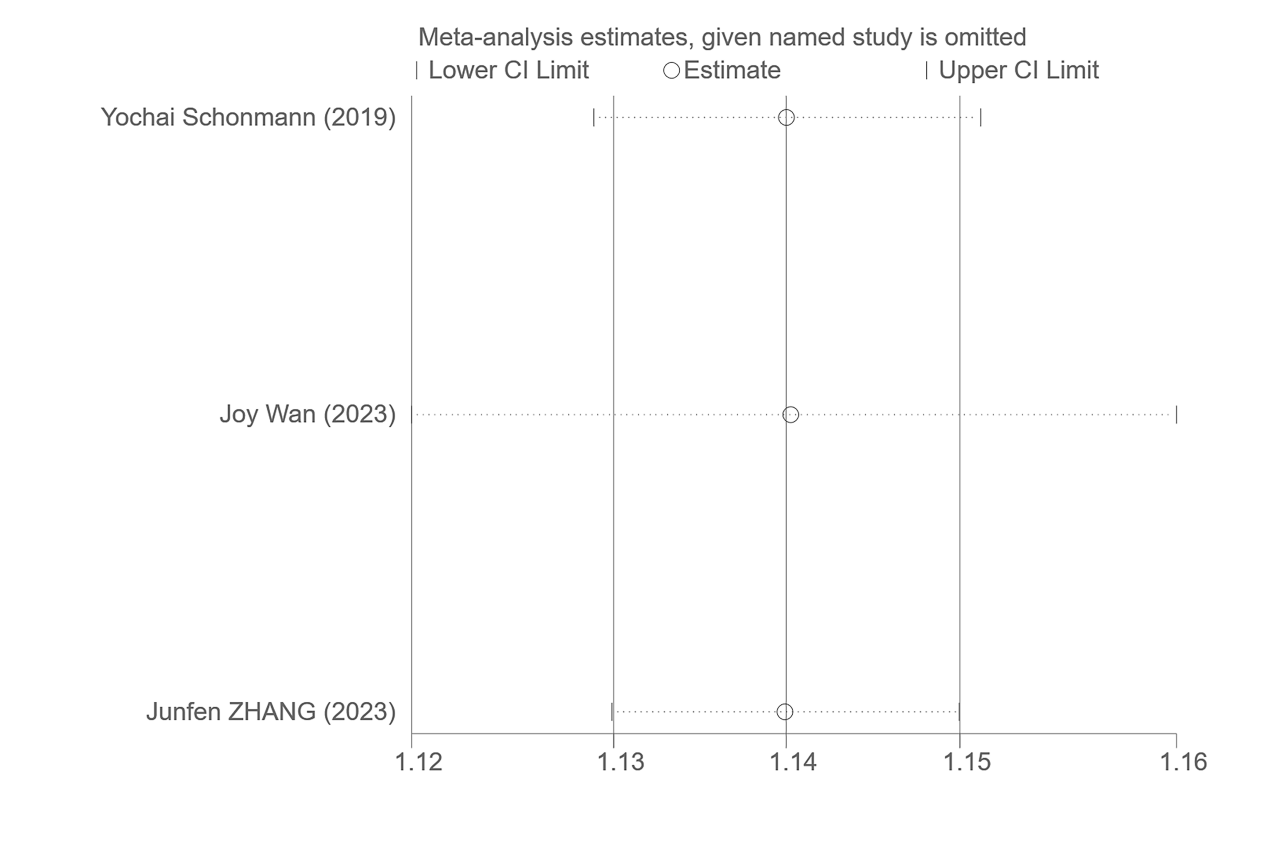


Supplementary Figure 20. Sensitivity analysis for the meta-analysis of depression in adults(HR).


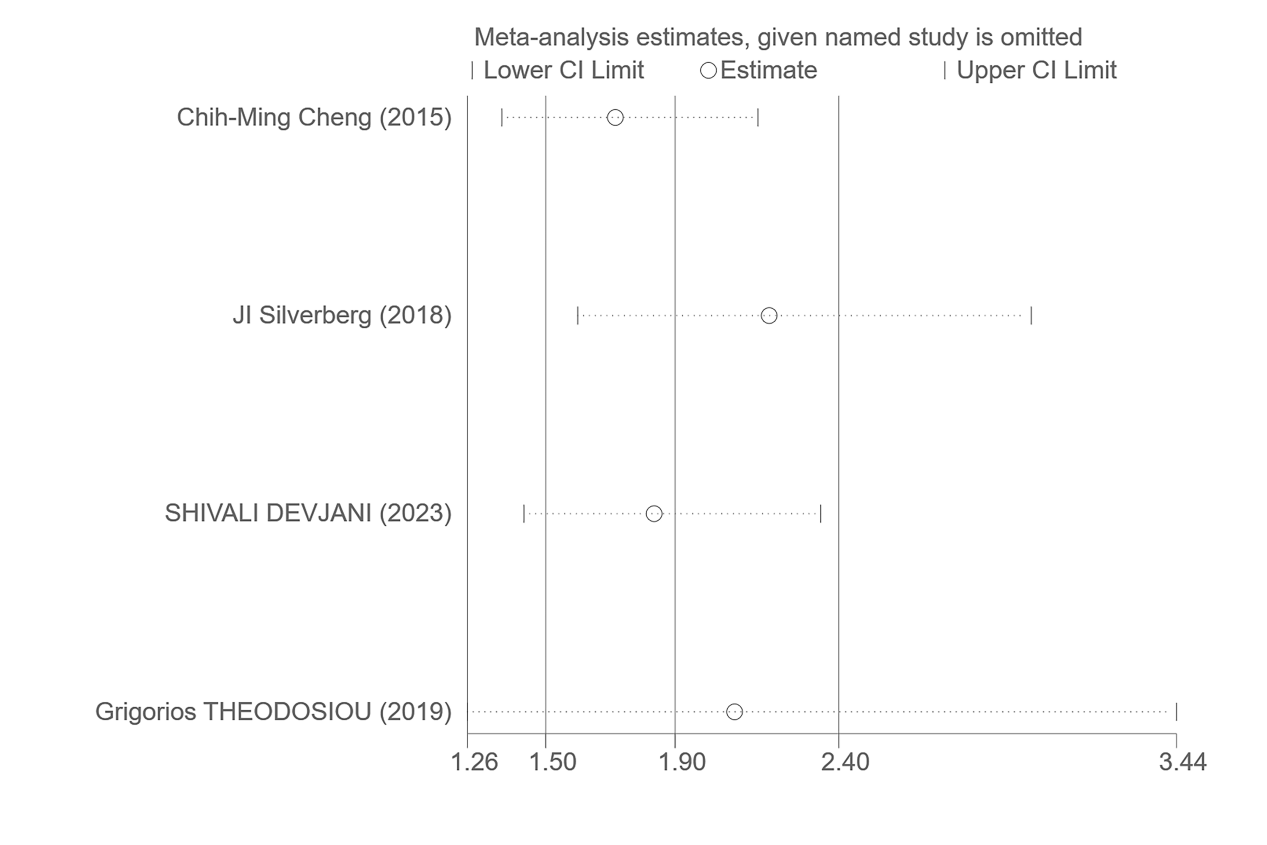


Supplementary Figure 21. Sensitivity analysis for the meta-analysis of anxiety in adults(OR).


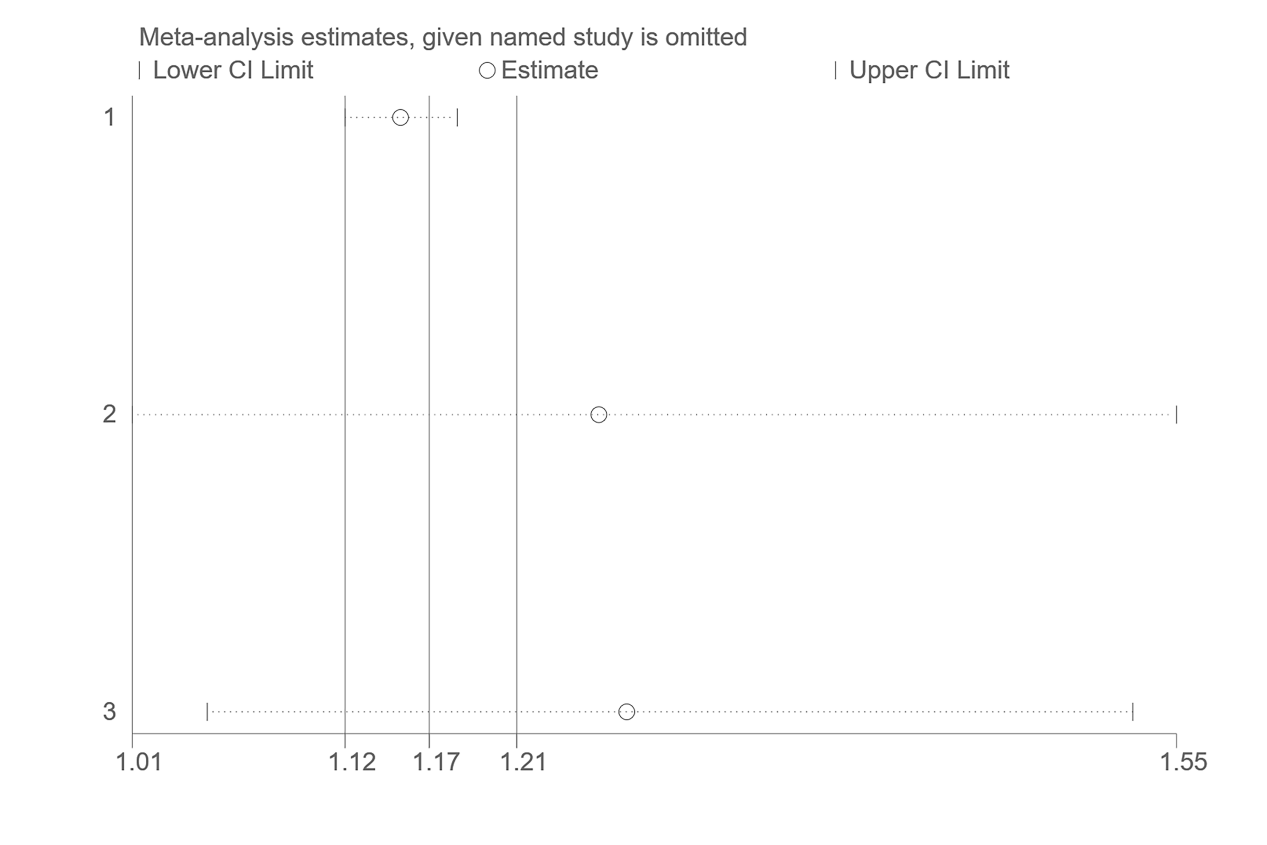
Supplementary Figure 22. Sensitivity analysis for the meta-analysis of anxiety in adults(HR).
