# Supplementary material for: Age differences in psychological comorbidity in atopic dermatitis: a systematic review and meta-analysis
Source: Front Public Health. 2026 Jul 7;14:1818252. doi: 10.3389/fpubh.2026.1818252 (PMC13384835; doi:10.3389/fpubh.2026.1818252)
Supplement: Supplementary file 2 [file Table_1.doc]

**Supplementary**

**Table 1-3:** **Details of the Literature Search Strategy**

**Table 1** PubMed

| Search | Query | Results |
| --- | --- | --- |
| 1 | ((((((((((((((((((((((((neuropsychiatric disorder[Title/Abstract]) OR (neurodevelopmental disorder[Title/Abstract])) OR (mental disease[Title/Abstract])) OR (autism[Title/Abstract])) OR (attention deficit disorder[Title/Abstract])) OR (Neurotoxicity, encephalopathy[Title/Abstract])) OR (altered mental status[Title/Abstract])) OR (confusion[Title/Abstract])) OR (cognitive impairment[Title/Abstract])) OR (behavioural changes[Title/Abstract])) OR (mood swing[Title/Abstract])) OR (agitation[Title/Abstract])) OR (hallucination[Title/Abstract])) OR (nervousness[Title/Abstract])) OR (aggression[Title/Abstract])) OR (insomnia[Title/Abstract])) OR (nightmares[Title/Abstract])) OR (dizziness[Title/Abstract])) OR (giddiness[Title/Abstract])) OR (disorientation[Title/Abstract])) OR (depression[Title/Abstract])) OR (anxiety[Title/Abstract])) OR (schizophrenia[Title/Abstract])) OR (bipolar disorder[Title/Abstract])) | [1,114,699](https://pubmed.ncbi.nlm.nih.gov/?term=((((((((((((((((((((((((neuropsychiatric+disorder[Title/Abstract])+OR+(neurodevelopmental+disorder[Title/Abstract]))+OR+(mental+disease[Title/Abstract]))+OR+(autism[Title/Abstract]))+OR+(attention+deficit+disorder[Title/Abstract]))+OR+(Neurotoxicity,+encephalopathy[Title/Abstract]))+OR+(altered+mental+status[Title/Abstract]))+OR+(confusion[Title/Abstract]))+OR+(cognitive+impairment[Title/Abstract]))+OR+(behavioural+changes[Title/Abstract]))+OR+(mood+swing[Title/Abstract]))+OR+(agitation[Title/Abstract]))+OR+(hallucination[Title/Abstract]))+OR+(nervousness[Title/Abstract]))+OR+(aggression[Title/Abstract]))+OR+(insomnia[Title/Abstract]))+OR+(nightmares[Title/Abstract]))+OR+(dizziness[Title/Abstract]))+OR+(giddiness[Title/Abstract]))+OR+(disorientation[Title/Abstract]))+OR+(depression[Title/Abstract]))+OR+(anxiety[Title/Abstract]))+OR+(schizophrenia[Title/Abstract]))+OR+(bipolar+disorder[Title/Abstract]))&ac=no&sort=relevance) |
| 2 | (((atopic dermatitis[Title/Abstract]) OR (eczema[Title/Abstract])) OR (chronic atopy[Title/Abstract])) | 52,472 |
| 3 | ((((((((((((((((((((((((neuropsychiatric disorder[Title/Abstract]) OR (neurodevelopmental disorder[Title/Abstract])) OR (mental disease[Title/Abstract])) OR (autism[Title/Abstract])) OR (attention deficit disorder[Title/Abstract])) OR (Neurotoxicity, encephalopathy[Title/Abstract])) OR (altered mental status[Title/Abstract])) OR (confusion[Title/Abstract])) OR (cognitive impairment[Title/Abstract])) OR (behavioural changes[Title/Abstract])) OR (mood swing[Title/Abstract])) OR (agitation[Title/Abstract])) OR (hallucination[Title/Abstract])) OR (nervousness[Title/Abstract])) OR (aggression[Title/Abstract])) OR (insomnia[Title/Abstract])) OR (nightmares[Title/Abstract])) OR (dizziness[Title/Abstract])) OR (giddiness[Title/Abstract])) OR (disorientation[Title/Abstract])) OR (depression[Title/Abstract])) OR (anxiety[Title/Abstract])) OR (schizophrenia[Title/Abstract])) OR (bipolar disorder[Title/Abstract])) AND (((atopic dermatitis[Title/Abstract]) OR (eczema[Title/Abstract])) OR (chronic atopy[Title/Abstract])) | 1,334 |

**Table 2** Cochrane Library

| **Search** | **Query** | **Results** |
| --- | --- | --- |
| #1 | (eczema):ti,ab,kw OR (atopic dermatitis):ti,ab,kw OR (chronic atopy):ti,ab,kw | 9,640 |
| #2 | (neuropsychiatric disorder):ti,ab,kw OR (neurodevelopmental disorder):ti,ab,kw OR (mental disease):ti,ab,kw OR (autism):ti,ab,kw OR (attention deficit disorder):ti,ab,kw | 41,702 |
| #3 | (Neurotoxicity, encephalopathy):ti,ab,kw OR (altered mental status):ti,ab,kw OR (confusion):ti,ab,kw OR (cognitive impairment):ti,ab,kw OR (behavioural changes):ti,ab,kw | 38,428 |
| #4 | (mood swing):ti,ab,kw OR (agitation):ti,ab,kw OR (hallucination):ti,ab,kw OR (nervousness):ti,ab,kw OR (aggression):ti,ab,kw | 13,450 |
| #5 | (insomnia):ti,ab,kw OR (nightmares):ti,ab,kw OR (dizziness):ti,ab,kw OR (giddiness):ti,ab,kw OR (disorientation):ti,ab,kw | 32,602 |
| #6 | (depression):ti,ab,kw OR (anxiety):ti,ab,kw OR (schizophrenia):ti,ab,kw OR (bipolar disorder):ti,ab,kw | 177,753 |
| #7 | #2 OR #3 OR #4 OR #5 OR #6 | 256,063 |
| #8 | #7 AND #1 | 503 |

**Table 3** Embase

| **Search** | **Query** | **Items found** |
| --- | --- | --- |
| #1 | 'neuropsychiatric disorder'/exp OR 'neuropsychiatric disorder' OR 'neurodevelopmental disorder':ab,ti OR 'mental disease':ab,ti OR 'autism':ab,ti OR 'attention deficit disorder':ab,ti OR 'neurotoxicity, encephalopathy':ab,ti OR 'altered mental status':ab,ti OR 'confusion':ab,ti OR 'cognitive impairment':ab,ti OR 'behavioural changes':ab,ti OR 'mood swing':ab,ti OR 'agitation':ab,ti | 3,434,725 |
| #2 | 'hallucination'/exp OR 'hallucination' OR 'nervousness':ab,ti OR 'aggression':ab,ti OR 'insomnia':ab,ti OR 'nightmares':ab,ti OR 'dizziness':ab,ti OR 'giddiness':ab,ti OR 'disorientation':ab,ti OR 'depression':ab,ti OR 'anxiety':ab,ti OR 'schizophrenia':ab,ti OR 'bipolar disorder':ab,ti | 1,275,814 |
| #3 | #1 OR #2 | 3,815,107 |
| #4 | 'atopic dermatitis'/exp OR 'atopic dermatitis' OR 'eczema':ab,ti OR 'chronic atopy':ab,ti | 97,343 |
| #5 | #3 AND #4 | 5,343 |
